# Supplementary material for: Induction of labour at 41 weeks or expectant management until 42 weeks: A systematic review and an individual participant data meta-analysis of randomised trials
Source: PLoS Med. 2020 Dec 8;17(12):e1003436. doi: 10.1371/journal.pmed.1003436 (PMC7723286; doi:10.1371/journal.pmed.1003436)

S1 Fig. Aggregate meta-analysis of studies comparing induction of labour with expectant management regarding perinatal mortality

Study or Subgroup	Induction Events	Induction Total	Expectancy Events	Expectancy Total	Weight	Peto Odds Ratio Peto, Fixed, 95% CI
Gelisen 2005	0	300	1	300	10.0%	0.14 [0.00, 6.82]
Keulen 2019	1	900	2	901	30.0%	0.51 [0.05, 4.94]
Wennerholm 2019	0	1381	6	1379	60.0%	0.13 [0.03, 0.67]
**Total (95% CI)**		**2581**		**2580**	**100.0%**	**0.20 [0.06, 0.70]**
Total events	1		9			

Heterogeneity: Chi² = 0.94, df = 2 (P = 0.63); I² = 0%
Test for overall effect: Z = 2.53 (P = 0.01)

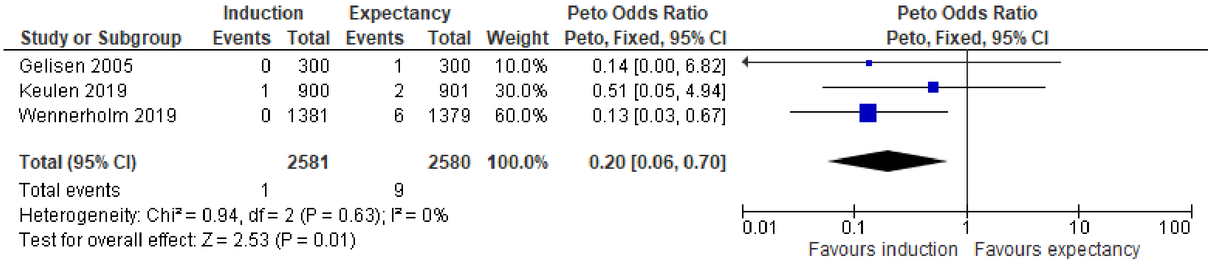

Supplement: S1 Fig — (PDF) [file pmed.1003436.s001.pdf]
