# Supplementary material for: Induction of labour at 41 weeks or expectant management until 42 weeks: A systematic review and an individual participant data meta-analysis of randomised trials
Source: PLoS Med. 2020 Dec 8;17(12):e1003436. doi: 10.1371/journal.pmed.1003436 (PMC7723286; doi:10.1371/journal.pmed.1003436)

S2 Fig. Aggregate meta-analysis of studies comparing induction of labour with expectant management regarding caesarean delivery

	Induction		Expectancy			Risk Ratio
**Study or Subgroup**	**Events**	**Total**	**Events**	**Total**	**Weight**	**M-H, Fixed, 95% CI**
Gelisen 2005	58	300	66	300	21.2%	0.88 [0.64, 1.20]
Keulen 2019	97	900	97	901	31.2%	1.00 [0.77, 1.31]
Wennerholm 2019	143	1381	148	1379	47.6%	0.96 [0.78, 1.20]
**Total (95% CI)**		**2581**		**2580**	**100.0%**	**0.96 [0.83, 1.11]**
Total events	298		311			
Heterogeneity: Chi² = 0.40, df = 2 (P = 0.82); I² = 0%						
Test for overall effect: Z = 0.57 (P = 0.57)						

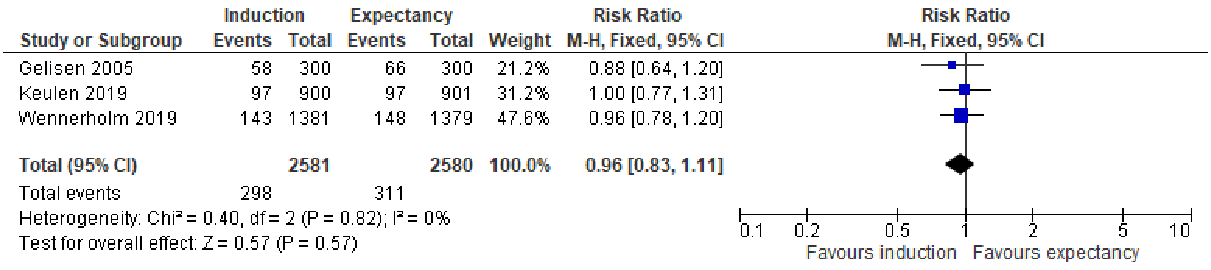

Supplement: S2 Fig — (PDF) [file pmed.1003436.s002.pdf]
