# Supplementary material for: Induction of labour at 41 weeks or expectant management until 42 weeks: A systematic review and an individual participant data meta-analysis of randomised trials
Source: PLoS Med. 2020 Dec 8;17(12):e1003436. doi: 10.1371/journal.pmed.1003436 (PMC7723286; doi:10.1371/journal.pmed.1003436)
Supplement: S1 Table — (PDF) [file pmed.1003436.s003.pdf]

S1 Table Baseline characteristics per trial in the populations included in the IPD-MA

Variable	SWEPIS Induction group (n=1381)	SWEPIS Expectant management group (n=1379)	INDEX Induction group (n=900)	INDEX Expectant management group (n=901)
Maternal age at randomisation (years)	**n=1381**	**n=1379**	**n=900**	**n=901**
Mean (standard deviation)	31.2 (4.7)	31.1 (4.5)	30.6 (4.8)	30.2 (4.6)
Age ≥35	303/1,381 (21.9)	279/1,379 (20.2)	176/900 (19.6)	152/901 (16.9)
Parity (includes stillbirths and live births)	**n=1381**	**n=1379**	**n=900**	**n=901**
Nulliparous	762/1,381 (55.2)	753/1,379 (54.6)	457/900 (50.8)	511/901 (56.7)
Multiparous	619/1,381 (44.8)	626/1,379 (45.4)	443/900 (49.2)	390/901 (43.3)
BMI at first antenatal visit	**n=1,275**	**n=1,265**	**n=877**	**n=888**
Mean (standard deviation)	24.9 (4.7	25.1 (4.9)	24.5 (4.3)	24.9 (4.7)
BMI ≥30	157/1,275 (12.3)	184/1,265 (14.5)	89/877 (10.1)	117/888 (13.2)
Higher professional education/university	789/1,221 (64.6)	780/1,242 (62.8)	286/900 (31.8)	322/901 (35.7)

Values are numbers (percentages) unless stated otherwise
