# Supplementary material for: Induction of labour at 41 weeks or expectant management until 42 weeks: A systematic review and an individual participant data meta-analysis of randomised trials
Source: PLoS Med. 2020 Dec 8;17(12):e1003436. doi: 10.1371/journal.pmed.1003436 (PMC7723286; doi:10.1371/journal.pmed.1003436)
Supplement: S2 Table — (PDF) [file pmed.1003436.s004.pdf]

S2 Table. Perinatal outcomes per trial in the populations included in the IPD-MA

Variable	SWEPIS Induction group (n=1,381)	SWEPIS Expectant management group (n=1,379)	INDEX Induction group (n=900)	INDEX Expectant management group (n=901)
**Primary outcome**				
Primary composite outcome*	8/1,381 (0.6)	15/1,379 (1.1)	2/900 (0.4)	8/901 (1.2)
**Subcomponents of primary composite outcome**				
Perinatal mortality†	0/1,381 (0.0)	6/1,379 (0.4)	1/900 (0.1)	2/901 (0.2)
Stillbirth	0/1,381 (0.0)	5/1,379 (0.4)	1/900 (0.1)	2/901 (0.2)
Neonatal mortality (Live births with mortality < 28 days)	0/1,381 (0.0)	1/1,374 (0.1)	0/899 (0.0)	0/899 (0.0)
Apgar score <4 at 5 minutes	3/1,381 (0.2)	1/1,374 (0.1)	0/899 (0.0)	3/899 (0.3)
Hypoxic ischemic encephalopathy (HIE) II-III	2/1,381 (0.1)	3/1,374 (0.2)	0/899 (0.0)	0/899 (0.0)
Intracranial haemorrhage	1/1,381 (0.1)	2/1,374 (0.1)	0/899 (0.0)	0/899 (0.0)
Neonatal convulsions	1/1,381 (0.1)	3/1,374 (0.2)	0/899 (0.0)	0/899 (0.0)
Meconium aspiration syndrome (MAS)	2/1,381 (0.1)	3/1,374 (0.2)	0/899 (0.0)	2/899 (0.2)
Tracheal intubation within first 72 hours	3/1,381 (0.2)	5/1,374 (0.4)	3/899 (0.3)	7/899 (0.8)
Obstetric brachial plexus injury	4/1,381 (0.3)	1/1,374 (0.1)	0/899 (0.0)	0/899 (0.0)
**Additional secondary neonatal outcome**				
Composite outcome with Apgar<7 at 5 minutes instead of <4	21/1,381 (1.5)	26/1,379 (1.9)	13/900 (1.4)	26/901 (2.9)
Admittance to neonatal care (all babies due to illness and not just protocol observation)	55/1,381 (4.0)	82/1,374 (6.0)	24/899 (2.7)	27/899 (3.0)
Admission to a neonatal care unit ≥4 days	21/1,381 (1.5)	36/1,374 (2.6)	3/899 (0.3)	10/899 (1.1)
Neonatal infection or Sepsis‡	12/1,381 (0.9)	22/1,374 (1.6)	37/899 (4.1)	37/899 (4.1)
Apgar score <7 at 5 minutes	18/1,381 (1.3)	16/1,374 (1.2)	11/899 (1.2)	23/899 (2.6)
Humerus fracture	0/1,381 (0.0)	0/1,379 (0.0)	0/900 (0.0)	1/901 (0.1)
Birth weight (g)	**n=1,381**	**n=1,379**	**n=900**	**n=901**
Mean (standard deviation)	3815 (409)	3875 (436)	3685 (417)	3742 (430)
Macrosomia (≥ 4500 g)	68/1,381 (4.9)	114/1,374 (8.3)	24/900 (2.4)	41/901 (4.3)
Small for gestational age§				
<3rd percentile	13/1,381 (0.9)	28/1,379 (2.0)	24/900 (2.7)	17/901 (1.9)
<10th percentile	94/1,381 (6.8)	103/1,379 (7.5)	75/900 (8.3)	85/901 (9.4)
Congenital anomaly¶	14/1,381 (1.0)	17/1,379 (1.2)	16/900 (1.8)	19/901 (2.1)
Boy	781/1,381 (56.6)	756/1,379 (54.8)	447/900 (49.7)	438/901 (48.6)

Values are numbers (percentages) unless stated otherwise.
*Including perinatal mortality, Apgar<4 at five minutes, HIE II-III, intracranial haemorrhage, neonatal convulsions, MAS, obstetrical brachial plexus injury, mechanical ventilation within 72 hours
†stillbirth and neonatal mortality (Live births with mortality < 28 days)
‡In the INDEX trial, neonates with suspected infection are also included
§ According to national gestational and sex specific references (18, 19)
¶ Minor birth anomalies according to EUROCAT excluded (17)
