# Supplementary material for: Induction of labour at 41 weeks or expectant management until 42 weeks: A systematic review and an individual participant data meta-analysis of randomised trials
Source: PLoS Med. 2020 Dec 8;17(12):e1003436. doi: 10.1371/journal.pmed.1003436 (PMC7723286; doi:10.1371/journal.pmed.1003436)
Supplement: S3 Table — (PDF) [file pmed.1003436.s005.pdf]

S3 Table. Delivery outcomes per trial in the populations included in the IPD-MA

Variable	SWEPIS Induction group (n=1,381)	SWEPIS Expectant management group (n=1,379)	INDEX Induction group (n=900)	INDEX Expectant management group (n=901)
**Gestational age at delivery (days)**	**n=1,381**	**n=1,379**	**n=900**	**n=901**
Median (interquartile range)	289 (283; 297)	292 (286; 297)	287 (285; 295)	289 (285; 296)
**Time from randomization to delivery (days)**	n=1,381	n=1,379	n=900	n=901
Mean (standard deviation)	1.76 (1.42)	4.66 (2.64)	2.06 (1.58)	4.16 (3.04)
**Onset of birth process**	**n=1,381**	**n=1,379**	**n=900**	**n=901**
Spontaneous	195/1,381 (14.1)	920/1,379 (66.7)	260/900(18.9)	664 (73.7)
Induction	1181/1,381 (85.5)	457/1,379 (33.1)	640 (71.1)	237 (26.3)
Scheduled caesarean delivery	5/1,381 (0.4)	2/1,379 (0.1)	0	0
Meconium stained amniotic fluid	233/1238 (18.8)	320/1126 (28.3)	147/900 (16.4)	205/901 (22.6)
Use of oxytocin*	907/1,381 (65.7)	722/1,379 (52.4)	533/900 (59.2)	355/901 (39.4)
**Mode of delivery**	**n=1,381**	**n=1,379**	**n=900**	**n=901**
Spontaneous vaginal delivery	1150/1,381 (83.3)	1140/1,379 (82.7)	710/900 (78.9)	696/901 (77.2)
Caesarean delivery	143/1,381 (10.4)	148/1,379 (10.7)	97/900 (10.8)	97/901 (10.8)
Operative vaginal delivery	88/1,381 (6.4)	91/1,379 (6.6)	93/900 (10.3)	108/901 (12.0)
**Indication for caesarean delivery**	**n=143**	**n=148**	**n=97**	**n=97**
Failure to progress†	79/143 (55.2)	83/148 (56.1)	41/97 (42.3)	39/97 (40.2)
Suspected foetal distress	41/143 (28.7)	36/148 (24.3)	24/97 (24.7)	21/97 (21.6)
Suspected foetal distress and failure to progress	6/143 (4.2)	10/148 (6.8)	11/97 (11.3)	11/97 (11.3)
Failed operative vaginal delivery	7/143 (4.9)	12/148 (8.1)	6/97 (6.2)	12/97 (12.4)
Other‡	10/143 (7.0)	7/148 (4.7)	15/97 (15.5)	14/97 (14.4)
**Indication for operative vaginal delivery**	**n=88**	**n=91**	**n=93**	**n=108**
Failure to progress§	50/88 (56.8)	49/91 (53.8)	39/93 (41.9)	49/108 (45.4)
Foetal distress	33/88 (37.5)	34/91 (37.4)	43/93 (46.2)	37/108 (34.3)
Foetal distress and Failure to progress	5/88 (5.7)	7/91 (7.7)	10/93 (10.8)	22/108 (20.4)
Maternal complication	0/88 (0.0)	1/91 (1.1)	1/93 (1.1)	0/108 (0.0)

Values are numbers (percentages) unless stated otherwise.
*Both induction and/or labour augmentation
†Including failed induction
‡Including scheduled due to e.g. undetected breech or transverse presentation/maternal indication
§Including maternal distress
