# Supplementary material for: Induction of labour at 41 weeks or expectant management until 42 weeks: A systematic review and an individual participant data meta-analysis of randomised trials
Source: PLoS Med. 2020 Dec 8;17(12):e1003436. doi: 10.1371/journal.pmed.1003436 (PMC7723286; doi:10.1371/journal.pmed.1003436)
Supplement: S4 Table — (PDF) [file pmed.1003436.s006.pdf]

S4 Table. Maternal outcomes per trial in the populations included in the IPD-MA

Variable	SWEPIS Induction group (n=1,381)	SWEPIS Expectant management group (n=1,379)	INDEX Induction group (n=900)	INDEX Expectant management group (n=901)
Pain treatment (use of epidural/spinal/opiates)*	733/1,381 (53.1)	675/1,379 (48.9)	420/900 (46.7)	383/901 (42.5)
Use of epidural anaesthesia	733/1,381 (53.1)	675/1,379 (48.9)	265/900 (29.4)	231/901 (25.6)
Use of opiates	0/1,381 (0.0)	0/1,379 (0.0)	184/900 (20.4)	173/901 (19.2)
Fever during labour	127/1,381 (9.2)	105/1,379 (7.6)	50/900 (5.6)	46/901 (5.1)
Antibiotics during labour	248/1,381 (18.0)	262/1,379 (19.0)	48/900 (5.3)	35/901 (3.9)
Therapy	95/1,381 (6.9)	80/1,379 (5.8)	33/900 (3.7)	24/901 (2.7)
Prophylaxis	153/1,381 (11.1)	182/1,379 (13.2)	15/900 (1.7)	11/901 (1.3)
Episiotomy†	89/1,381 (6.4)	84/1,379 (6.1)	239/900 (26.6)	251/901 (27.9))
Perineal lacerations III-IV‡	40/1,381 (2.9)	50/1,379 (3.6)	28/900 (3.1)	31/901 (3.4)
Postpartum haemorrhage (>1000 ml) §	131/1377 (9.5)	145/1375 (10.5)	77/900 (9.1)	59/901 (8.0)
Postpartum haemorrhage (>2000 ml) §	27/1,381 (2.0)	18/1,379 (1.3)	15/900 (3.0)	16/901 (2.6)
Retained placenta (all)	52/1,381 (3.8)	57/1,379 (4.1)	41/900 (4.6)	33/901 (3.7)
Retained placenta with haemorrhage >1000 ml	36/1,381 (2.6)	36/1,379 (2.6)	26/900 (2.9)	25/901 (2.8)
Retained placenta with haemorrhage ≤1000 ml	16/1,381 (1.2)	21/1,379 (1.5)	15/900 (1.7)	8/901 (0.9)
Hypertensive disorders¶	19/1,381 (1.4)	42/1,379 (3.0)	7/900 (0.6)	24/901 (1.6)
Maternal venous thromboembolism	0/1,381 (0.0)	1/1,379 (0.1)	0/900 (0.0)	0/901 (0.0)
Maternal admission to intensive care unit	2/1,381(0.1)	0/1,381 (0.0)	3/900 (0.3)	2/901 (0.2)
Maternal death	0/1,381 (0.0)	0/1,379 (0.0)	0/900 (0.0)	0/901 (0.0)

Values are numbers (percentages) unless stated otherwise.
*In the INDEX trial a combination of epidural and opiates was possible
†With and without perineal lacerations III-IV
‡With and without episiotomy
§Based on measured blood loss and not International Classification of Diseases 10th Revision codes reported
¶Hypertensive disorders of pregnancy including eclampsia and HELLP
