# Supplementary material for: Induction of labour at 41 weeks or expectant management until 42 weeks: A systematic review and an individual participant data meta-analysis of randomised trials
Source: PLoS Med. 2020 Dec 8;17(12):e1003436. doi: 10.1371/journal.pmed.1003436 (PMC7723286; doi:10.1371/journal.pmed.1003436)
Supplement: S5 Table — (PDF) [file pmed.1003436.s007.pdf]

S5 Table. Primary outcome: severe adverse perinatal outcome, perinatal mortality and caesarean delivery per subgroup in the population included in the IPD-MA.

Variable	Induction group	Expectant management group	Relative Risk (95% Confidence Interval)	p-value	Risk difference per 10,000 (95% Confidence Interval)	Inter-action p-value
**Severe adverse perinatal outcome ***						
**Parity**						0.01
Nulliparous	4/1,219 (0.3)	20/1,264 (1.6)	0.20 (0.07; 0.60)	0.004	-127 (-204; -50)	
Multiparous	6/1,062 (0.6)	3/1,016 (0.3)	1.93 (0.48; 7.72)	0.35	27 (-29; 84)	
**Maternal age (Years)**						0.23
<35 years	8/1,802(0.4)	14/1,849 (0.8)	0.59 (0.25; 1.39)	0.23	-31 (-82; 19)	
≥35 years	2/479 (0.4)	9/431 (2.1)	0.20 (0.04; 0.93)	0.04	-166 (-308; -25)	
**Body mass index (Kg/m²)**						0.25
<30	8/1,906 (0.4)	19/1,852 (1.0)	0.32 (0.13; 0.81)	0.02	-66 (-117; -15)	
≥30	3/246 (1.2)	4/301 (1.3)	0.91 (0.21; 4.02)	0.90	-12 (-202; 177)	
**Fetal sex**						0.10
Boy	5/1,228 (0.4)	18/1,194 (1.5)	0.27 (0.10; 0.72)	0.01	-110 (-187; -33)	
Girl	5/1,053 (0.5)	5/1,086 (0.5)	1.03 (0.30; 3.56)	0.96	2 (-56; 59)	
**Perinatal mortality†**						
**Parity**						NE
Nulliparous	0/1,219 (0.0)	7/1,264 (0.6)	0.14 (0.03; 0.60)‡	0.01	-56 (-98; -15)	
Multiparous	1/1,062 (0.1)	1/1,016 (0.1)	0.88 (0.05; 14.17)‡	0.93	-1 (-28; 25)	
**Maternal age (Years)**						NE
<35 years	1/1,802 (0.1)	4/1,849 (0.2)	0.31 (0.05; 1.78)‡	0.19	-16 (-40; 8)	
≥35 years	0/479 (0.0)	4/431 (0.9)	0.12 (0.02; 0.87‡	0.04	-93 (-178; -7)	
**Body mass index (Kg/m²)**						NE
<30	1/1,906 (0.1)	5/1,852(0.3)	0.26 (0.05; 1.27)‡	0.10	-22 (-47; 4)	
≥30	0 /246 (0.0)	3/301 (1.0)	0.16 (0.02; 1.57)‡	0.12	-100 (-224; 24)	
**Fetal sex**						NE
Boy	1/1,228 (0.1)	4/1,194 (0.3)	0.29 (0.05; 1.69)‡	0.17	-25 (-61; 11)	
Girl	0/1,053 (0.0)	4/1,086 (0.4)	0.14 (0.02; 0.99)‡	0.05	-37 (-73; -0)	
**Caesarean delivery**						
**Parity**						0.88
Nulliparous	219/1,219 (18.0)	226/1,264 (17.9)	1.01 (0.85; 1.19)	0.95	10 (-292; 312)	
Multiparous	21/1,062 (2.0)	19/1,016 (1.9)	1.05 (0.57; 1.95)	0.87	10 (-108; 128	
**Maternal age (Years)**						0.07
<35 years	194/1,802 (10.8)	188/1,849 (10.2)	1.06 (0.88; 1.28)	0.56	60 (-139; 258	
≥35 years	46/479 (9.6)	57/431 (13.2)	0.73 (0.50; 1.05)	0.09	-364 (-775; 48)	
**Body mass index (Kg/m²)**						0.32
<30	202/1,906 (10.6)	188/1,852 (10.2)	1.04 (0.87; 1.26)	0.65	45 (-150; 240)	
≥30	28/246 (11.4)	42 (14.0)	0.82 (0.52; 1.28)	0.38	-254 (-816; 309)	
**Fetal sex**						0.22
Boy	141/1,228 (11.5)	153/1,194 (12.8)	0.90 (0.72; 1.11)	0.31	-133 (-393; 127)	
Girl	99/1,053 (9.4)	92/1,086 (8.5)	1.11 (0.85; 1.45)	0.45	94 (-148; 335)	
**Use of Oxytocin**						
**Parity**						<0.001
Nulliparous	925 (75.9)	817 (64.6)	1.15 (1.10; 1.21)	<0.001	1083 (730; 1437)	
Multiparous	515 (48.5)	260 (25.6)	1.89 (1.68; 2.14)	<0.001	2304 (1900; 2708)	

Values are numbers (percentages) unless stated otherwise. Relative risk is adjusted for RCT. P-value correspond to the method used to calculate the relative risk/odds ratio. NE= not estimable due to low number of events (total of nine)

*Composite of stillbirth, neonatal mortality, Apgar<4 at five minutes, HIE II-III, intracranial hemorrhage, neonatal convulsions, meconium aspiration syndrome, obstetric brachial plexus injury and mechanical ventilation within 72 hours
†Stillbirth and neonatal mortality (live births with mortality <28 days)
‡Peto odds ratio
