# Supplementary material for: Induction of labour at 41 weeks or expectant management until 42 weeks: A systematic review and an individual participant data meta-analysis of randomised trials
Source: PLoS Med. 2020 Dec 8;17(12):e1003436. doi: 10.1371/journal.pmed.1003436 (PMC7723286; doi:10.1371/journal.pmed.1003436)
Supplement: S6 Table — (PDF) [file pmed.1003436.s008.pdf]

S6 Table. Data availability contact information

Trial	Contact information
Keulen et al., 2019	Dr Madelon van Wely<br>Center for Reproductive Medicine<br>Amsterdam UMC, Location AMC, Room Q3-174<br>Meibergdreef 9, 1105AZ Amsterdam<br>phone: +31 642238278<br>e-mail:m.vanwely@amsterdamumc.nl
Wennerholm et al., 2019	Senior Professor, Senior Consultant<br>Department of Pediatrics<br>University of Gothenburg<br>Queen Silvia Children's Hospital<br>SE-416 85 Gothenburg, SWEDEN<br><br>phone +46 (0)31 343 46 12<br>phone +46 (0)31 40 10 79 (home)<br>mobile phone +46 (0)705 37 46 12<br>e-mail goran.wennergren@pediat.gu.se
