# Supplementary material for: Induction of labour at 41 weeks or expectant management until 42 weeks: A systematic review and an individual participant data meta-analysis of randomised trials
Source: PLoS Med. 2020 Dec 8;17(12):e1003436. doi: 10.1371/journal.pmed.1003436 (PMC7723286; doi:10.1371/journal.pmed.1003436)
Supplement: S2 Text — (PDF) [file pmed.1003436.s010.pdf]

S2 Text. PICO, study selection, search strategies, and references of excluded studies

Question at issue:

PICO: (*P=Patient I=Intervention C=Comparison O=Outcome*)

P - Women with a singleton, uncomplicated (as defined by authors) pregnancy with a child in cephalic presentation. Gestational age had to be confirmed with early ultrasound.

I - Strategy to induce labour at 41+ 0 - 2.

C - Strategy of expectant management (with various regimes of fetal surveillance) and induction of labour at 42+ 0 - 1.

O - Primary outcome ’severe adverse perinatal outcome’ a composite of perinatal mortality and severe neonatal morbidity. Perinatal mortality defined as stillbirth, or neonatal mortality of live births with death between day 0 and 28 (deaths due to accidents were excluded). Severe neonatal morbidity defined as a composite of I) 5 minute Apgar score <4, II) hypoxic-ischemic encephalopathy II-III (asphyxia/encephalopathy in need of therapeutic cooling), III) intracranial haemorrhage (intracranial haemorrhage based on radiological findings with ultrasound of the brain, computed tomography (CT) or magnetic resonance imaging (MRI), including intraventricular haemorrhage), VI) neonatal convulsions (seizures with electroencephalography [EEG]/amplitude-EEG confirmation, V) seizures without EEG/amplitude EEG confirmation and silent seizures [EEG/diagnosis]), VI) MAS (respiratory distress after birth in the presence of meconium stained amniotic fluid with need of mechanical ventilation), VII) mechanical ventilation within the first 72 hours (with laryngeal tube and ventilator machine) and/or VIII) obstetric brachial plexus injury.

Secondary perinatal outcomes consist of all individual components of the composite outcome separately including stillbirth and neonatal mortality. Additional secondary outcomes are: the composite outcome with Apgar <7 instead of Apgar <4, admission to neonatal care (medium or intensive care unit), admission to neonatal care ≥ 4 days mimicking neonatal care for infants in need for longer treatment and more extensive observation, mean birthweight, small for gestational age (SGA) according to national birthweight curves (<10th percentile and <3rd percentile) [21, 22], macrosomia (≥4500g), 5 minutes Apgar score <7, , infection/sepsis (clinical suspected findings or proved positive blood culture and antibiotic treatment), meconium stained amniotic fluid, humerus fracture and congenital anomalies (any congenital anomalies after excluding minor congenital anomalies according to the European Surveillance of Congenital Anomalies [EUROCAT]).

Secondary maternal outcomes includes interval from randomisation to delivery, gestational age at time of delivery, onset of labour (spontaneously or IOL), oxytocin during labour (for IOL and/or augmentation), pain treatment (epidural anaesthesia/spinal anaesthesia/opiates), mode of delivery (spontaneous vaginal delivery, assisted vaginal delivery or caesarean section with indication for intervention), episiotomy, perineal lacerations III-IV (with or without episiotomy), postpartum haemorrhage (>1000 ml and >2000 ml), fever during labour (≥ 38°C), antibiotics during labour (prophylaxis or therapy), manual removal of placenta (with or without haemorrhage >1000 ml), hypertensive disorders of pregnancy including eclampsia and HELLP, maternal deep vein thrombosis or pulmonary embolism, admission to intensive care unit and maternal death up to 42 days after delivery (deaths due to accidents excluded).

Eligibility criteria

Study design: Randomised controlled trials

Language: no restriction

A first search was made with language limitation English, Swedish, Norwegian, Danish and Dutch. An additional search using the same search strategy including all languages except the ones mentioned above with no language limitation was performed.

Search strategies

Total number of hits: 1552 in first search and 112 in the additional search

Number of hits after removal of duplicates: 1012 in the first search and 88 in the additional search.

Database: PubMed

Date: 21 Feb 2020

No. of results: 736 in first search and 52 in the additional search

Search	Query	Items found
**#19**	**Search #11 NOT #12 Filters: Publication date from 2011/10/01; Swedish; Norwegian; English; Danish**	**736**
#18	Search #11 NOT #12 Filters: Swedish; Norwegian; English; Danish	1922
#13	Search #11 NOT #12	2345
#12	Search Editorial[ptyp] OR Letter[ptyp] OR Comment[ptyp]	1808560
#11	Search #7 NOT #10	2396
#10	Search #8 OR #9	4999082
#9	Search animal[ti] OR animals[ti] OR rat[ti] OR rats[ti] OR mouse[ti] OR mice[ti] OR rodent[ti] OR rodents[ti] OR dog[ti] OR dogs[ti] OR cat[ti] OR cats[ti] OR hamster[ti] OR hamsters[ti] OR rabbit[ti] OR rabbits[ti] OR swine[ti] OR murine[ti]	1854065

#8	Search ((animals[mh]) NOT (animals[mh] AND humans[mh]))	4670734
#7	Search #3 AND #6	2790
#6	Search #4 OR #5	35866
#5	Search "pregnancy, prolonged"[MeSH Terms]	2644
#4	Search (full-term OR fullterm OR post-date OR postdate OR post-term OR postterm OR late term[tiab] OR beyond term[tiab] OR 41 weeks[tiab] OR 42 weeks[tiab] OR prolonged) AND (pregnancy OR pregnancies OR delivery OR deliveries)	35866
#3	Search #1 OR #2	500889
#2	Search "Labor, Induced"[Mesh]	9261
#1	Search (induced[tiab] AND (labor[tiab] OR labour[tiab])) OR induction[tiab] OR (expectant[tiab] AND management[tiab])	497710

Database: Embase 1974 to 2020 February 19 (OvidSP)

Date: 21 Feb 2020

No. of results: 435 in the first search and 55 in the additional search

**Search**	**Query**	**Items found**
1	((induced and (labor or labour)) or induction or (expectant and management)).ab,kw,ti.	642944
2	exp labor induction/	13474
3	1 or 2	648139
4	((full-term or fullterm or post-date or postdate or post-term or postterm or late term or beyond term or 41 weeks or 42 weeks or prolonged) adj4 (pregnancy or pregnancies or delivery or deliveries)).ab,kw,ti.	9160
5	prolonged pregnancy/	2140
6	4 or 5	10103
7	3 and 6	1723

8	(animal not (animal and human)).sh.	1059747
9	(animal or animals or rat or rats or mouse or mice or rodent or rodents or dog or dogs or cat or cats or hamster or hamsters or rabbit or rabbits or swine or murine).ti.	1963900
10	8 or 9	2788535
11	7 not 10	1691
**12**	**limit 11 to ((danish or english or norwegian or swedish) and yr="2011 - Current" and (article or article in press or conference paper or "review"))**	**435**

Database: CINAHL, APA PsycInfo via EBSCOhost

Date: 21 Feb 2020

No. of results: 203 (after removal of duplicates between databases) in the first search and 5 in the additional search

Search	Query	Items found
**S5**	**S1 AND S2**<br>**Limitation – Publication date: 20110101-20191231**	**208**
S4	S1 AND S2	208
S3	S1 AND S2	316
S2	TI ((full-term OR fullterm OR post-date OR postdate OR post-term OR postterm OR "late term" OR "beyond term" OR "41 weeks" OR "42 weeks" OR prolonged) N4 (pregnancy OR pregnancies OR delivery OR deliveries)) OR AB ((full-term OR fullterm OR post-date OR postdate OR post-term OR postterm OR "late term" OR "beyond term" OR "41 weeks" OR "42 weeks" OR prolonged) N4 (pregnancy OR pregnancies OR delivery OR deliveries))	2,039
S1	TI ((induced AND (labor OR labour)) OR induction OR (expectant AND management)) OR AB ((induced AND (labor OR labour)) OR induction OR (expectant AND management))	59,504

Database: Cochrane Library

Date: 21 Feb 2020

No. of results: 178 (no language limitation in this search)

Cochrane reviews: 8

Trials: 170

Search	Query	Items found
#1	(((induced and (labor or labour)) or induction or (expectant and management))):ti,ab,kw (Word variations have been searched)	54478
#2	((full-term or fullterm or post-date or postdate or post-term or postterm or "late term" or "beyond term" or "41 weeks" or "42 weeks" or prolonged) NEAR/4 (pregnancy or pregnancies or delivery or deliveries)):ti,ab,kw (Word variations have been searched)	1296
#3	#1 AND #2	475
#4	(clinicaltrials or trialsearch):so	313883
**#5**	**#3 NOT #4 with Cochrane Library publication date Between Jul 2011 and Oct 2019**	**178**

Included studies:

Gelisen O, Caliskan E, Dilbaz S, Ozdas E, Dilbaz B, Ozdas E, et al. Induction of labor with three different techniques at 41 weeks of gestation or spontaneous follow-up until 42 weeks in women with definitely unfavorable cervical scores. Eur J Obstet Gynecol Reprod Biol. 2005;120(2):164-9.

Keulen JK, Bruinsma A, Kortekaas JC, van Dillen J, Bossuyt PM, Oudijk MA, et al. Induction of labour at 41 weeks versus expectant management until 42 weeks (INDEX): multicentre, randomised non-inferiority trial. BMJ. 2019;364:l344.

Wennerholm UB, Saltvedt S, Wessberg A, Alkmark M, Bergh C, Wendel SB, et al. Induction of labour at 41 weeks versus expectant management and induction of labour at 42 weeks (SWEdish Post-term Induction Study, SWEPIS): multicentre, open label, randomised, superiority trial. BMJ (Clinical research ed). 2019;367:l6131.

Excluded articles with reason

**Article**	**Reason for exclusion**
Akuamoah-Boateng J, Spencer R. Woman-centered care: Women's experiences and perceptions of induction of labor for uncomplicated post-term pregnancy: A systematic review of qualitative evidence. Midwifery. 2018;67:46-56	Other study design, not RCT. Systematic review of women’s experiences and perceptions of labour. Other gestational age.
Augensen K, Bergsjo P, Eikeland T, Askvik K, Carlsen J. Randomised comparison of early versus late induction of labour in post-term pregnancy. British medical journal (Clinical research ed). 1987;294(6581):1192-5	Other research question.
Beckmann M, Gibbons K, Flenady V, Kumar S. Induction of labour using prostaglandin E2 as an inpatient versus balloon catheter as an outpatient: a multicentre randomised controlled trial. BJOG : an international journal of obstetrics and gynaecology. 2019	Other research question.
Bendix JM, Friis Petersen J, Andersen BR, Bodker B, Lokkegaard EC. Induction of labor with high- or low-dosage oral misoprostol-A Danish descriptive retrospective cohort study 2015-16. Acta obstetricia et gynecologica Scandinavica. 2020;99(2):222-30	Other study design, not RCT. Other gestational age in intervention and comparison group.
Benito Reyes V, Hurtado Mendoza R, Rodriguez Rodriguez F, Reyes Suarez D, Alvarez Leon EE, Garcia Hernandez JA. Elective termination versus expectant management in prolonged pregnancy: A prospective study of 200 pregnant women. [Spanish] Finalizacion electiva versus manejo expectante en el control de la gestacion prolongada: Estudio prospectivo de 200 gestantes. Progresos de Obstetricia y Ginecologia. 2010;53(11):446-53.	Other study design, not RCT.
Bergsjö P, Huang GD, Yu SQ, Gao ZZ, Bakketeig LS. Comparison of induced versus non-induced labor in post-term pregnancy. A randomized prospective study. Acta obstetricia et gynecologica Scandinavica. 1989;68(8):683-7	Other gestational age in one or both comparison groups.

Boshomane JM, Sebitloane HM. Factors associated with successful induction of labour with oral misoprostol in term or post-term pregnancies. Obstetrics and Gynaecology Forum. 2019;29(3):25-8	Other study design, not RCT. Other research question.
Carlhall S, Kallen K, Blomberg M. The effect of maternal body mass index on duration of induced labor. Acta obstetricia et gynecologica Scandinavica. 2019	Other study design, not RCT. Other research question.
Chakravarti S, Goenka B. Conservative policy of induction of labor in uncomplicated postdated pregnancies [abstract]. XVI FIGO World Congress of Obstetrics & Gynecology; 2000 Sept 3-8; Washington DC, USA. 2000:62	Other gestational age in one or both comparison groups. Other publication type (congress abstract).
Chanrachakul B, Herabutya Y. Postterm with favorable cervix: is induction necessary? European journal of obstetrics, gynecology, and reproductive biology. 2003;106(2):154-7	Other gestational age in one or both comparison groups.
Cheng YW, Kaimal AJ, Snowden JM, Nicholson JM, Caughey AB. Induction of labor compared with expectant management in low-risk women and associated perinatal outcomes. Am J Obstet Gynecol. 2012a;207(6):502.e1-8	Other study design, not RCT. Other gestational age in intervention and comparison group.
Cheng YW, Sparks TN, Laros RK, Jr., Nicholson JM, Caughey AB. Impending macrosomia: will induction of labour modify the risk of caesarean delivery? BJOG. 2012b;119(4):402-9	Other study design, not RCT. Other gestational age in intervention and comparison group.
Coates D, Makris A, Catling C, Henry A, Scarf V, Watts N, et al. A systematic scoping review of clinical indications for induction of labour. PloS one. 2020;15(1):e0228196	Other study design, not RCT. Other research question. A systematic scoping review of quantitative studies on indication for induction.
Danilack VA, Dore DD, Triche EW, Muri JH, Phipps MG, Savitz DA. The effect of labour induction on the risk of caesarean delivery: using propensity scores to control confounding by indication. BJOG. 2016a;123(9):1521-9	Other study design, not RCT. Other gestational age in intervention and comparison group.
Danilack VA, Triche EW, Dore DD, Muri JH, Phipps MG, Savitz DA. Comparing expectant management and spontaneous labor approaches in studying the effect of labor induction on cesarean delivery. Ann Epidemiol. 2016b;26(6):405-11.e1	Other study design, not RCT. Other gestational age in intervention and comparison group.
Direkvand-Moghadam A, Delpisheh A, Yousefinezhad A, Jaafarpour M, Ashrafi Hafez A. Effect of Oral Propranolol on Labor Induction in Nulliparous Women With Prolonged Pregnancy. Advances in Nursing & Midwifery. 2013;23(82):17-23	Other research question.
Dyson DC, Miller PD, Armstrong MA. Management of prolonged pregnancy: induction of labor versus antepartum fetal testing. American journal of obstetrics and gynecology. 1987;156(4):928-34	Other gestational age in one or both comparison groups.
Grivell RM, Reilly AJ, Oakey H, Chan A, Dodd JM. Maternal and neonatal outcomes following induction of labor: a cohort study. Acta Obstet Gynecol Scand. 2012;91(2):198-203	Other study design, not RCT. Other gestational age in intervention and comparison groups.

Grunewald C, Hakansson S, Saltvedt S, Kallen K. Significant effects on neonatal morbidity and mortality after regional change in management of post-term pregnancy. Acta Obstet Gynecol Scand. 2011;90(1):26-32	Other study design, not RCT. Other population/comparison. Includes multiple pregnancies. Other strategy.
Hannah ME, Hannah WJ, Hellmann J, Hewson S, Milner R, Willan A. Induction of labor as compared with serial antenatal monitoring in post-term pregnancy. A randomized controlled trial. The Canadian Multicenter Post-term Pregnancy Trial Group. The New England journal of medicine. 1992;326(24):1587-92	Other gestational age in one or both comparison groups.
Hedegaard M, Lidegaard O, Skovlund CW, Morch LS, Hedegaard M. Reduction in stillbirths at term after new birth induction paradigm: results of a national intervention. BMJ Open. 2014;4(8):e005785	Other study design, not RCT. Other gestational age in intervention and comparison groups.
Heimstad R, Skogvoll E, Mattsson LA, Johansen OJ, Eik-Nes SH, Salvesen KA. Induction of labor or serial antenatal fetal monitoring in postterm pregnancy: a randomized controlled trial. Obstetrics and gynecology. 2007a;109(3):609-17	Other gestational age in one or both comparison groups.
Heimstad R, Romundstad PR, Hyett J, Mattsson LA, Salvesen KA. Women's experiences and attitudes towards expectant management and induction of labor for post-term pregnancy. Acta obstetricia et gynecologica Scandinavica. 2007b;86(8):950-6	Other gestational age in one or both comparison groups. Other research question.
Henry GR. A controlled trial of surgical induction of labour and amnioscopy in the management of prolonged pregnancy. The Journal of obstetrics and gynaecology of the British Commonwealth. 1969;76(9):795-8	Other gestational age in one or both comparison groups.
Herabutya Y, Prasertsawat PO, Tongyai T, Isarangura Na Ayudthya N. Prolonged pregnancy: the management dilemma. International journal of gynaecology and obstetrics: the official organ of the International Federation of Gynaecology and Obstetrics. 1992;37(4):253-8	Other gestational age in one or both comparison groups.
Hutcheon JA, Harper S, Strumpf EC, Lee L, Marquette G. Using inter-institutional practice variation to understand the risks and benefits of routine labour induction at 41(+0) weeks. BJOG. 2015;122(7):973-81	Other study design, not RCT. Other comparison.
James C, George SS, Gaunekar N, Seshadri L. Management of prolonged pregnancy: a randomized trial of induction of labour and antepartum fetal monitoring. The National medical journal of India. 2001;14(5):270-3	Other gestational age in one or both comparison groups.
Kaimal AJ, Little SE, Odibo AO, Stamilio DM, Grobman WA, Long EF, et al. Cost-effectiveness of elective induction of labor at 41 weeks in nulliparous women. Am J Obstet Gynecol. 2011;204(2):137.e1-.e9	Other study design, not RCT. Data on cost effectiveness, included in cost effectiveness analysis, included as “other references.”
Keulen JKJ, Bruinsma A, Kortekaas JC, van Dillen J, van der Post JAM, de Miranda E. Timing induction of labour at 41 or 42 weeks? A closer look at time frames of comparison: A review. Midwifery. 2018;66:111-8	Other study design, not RCT. Systematic review, 22 RCTs. Overlap with HTA report from 2012 (Wennerholm et al., 2012) and the Cochrane reviews Gulmezoglu et al., 2012 and Middleton et al., 2018 with focus on 41-42 gestational week time frame. No further trials identified with gestational age according to PICO.

Knight HE, Cromwell DA, Gurol-Urganci I, Harron K, van der Meulen JH, Smith GCS. Perinatal mortality associated with induction of labour versus expectant management in nulliparous women aged 35 years or over: An English national cohort study. PLoS Med. 2017;14(11):e1002425	Other study design, not RCT. Other intervention and comparison groups.
Kortekaas JC, Kazemier BM, Keulen JKJ, Bruinsma A, Mol BW, Vandenbussche F, et al. Risk of adverse pregnancy outcomes of late- and postterm pregnancies in advanced maternal age: a national cohort study. Acta obstetricia et gynecologica Scandinavica. 2020	Other study design, not RCT. Other research question.
Lindegren L, Stuart A, Herbst A, Kallen K. Improved neonatal outcome after active management of prolonged pregnancies beyond 41(+2) weeks in nulliparous, but not among multiparous women. Acta Obstet Gynecol Scand. 2017;96(12):1467-74	Other study design, not RCT. Other intervention and comparison groups. No data on pregnancy dating.
Liu S, Joseph KS, Hutcheon JA, Bartholomew S, Leon JA, Walker M, et al. Gestational age-specific severe maternal morbidity associated with labor induction. Am J Obstet Gynecol. 2013;209(3):209.e1-8	Other study design, not RCT. Other intervention and comparison groups.
Lou S, Hvidman L, Uldbjerg N, Neumann L, Jensen TF, Haben JG, et al. Women's experiences of postterm induction of labor: A systematic review of qualitative studies. Birth. 2019;46(3):400-10	Other study design, not RCT. Systematic review, other research question, other gestational age.
Martin JN, Jr., Sessums JK, Howard P, Martin RW, Morrison JC. Alternative approaches to the management of gravidas with prolonged-postterm-postdate pregnancies. Journal of the Mississippi State Medical Association. 1989;30(4):105-11	Other gestational age in one or both comparison groups.
Maeder AB, Park CG, Vonderheid SC, Bell AF, Carter CS, McFarlin BL. Maternal and system characteristics, oxytocin administration practices, and cesarean birth rate. Birth (Berkeley, Calif). 2020;30	Other study design, not RCT. Other research question.
Marquette GP, Hutcheon JA, Lee L. Predicting the spontaneous onset of labour in post-date pregnancies: a population-based retrospective cohort study. J Obstet Gynaecol Can. 2014;36(5):391-9	Other study design, not RCT. Other outcome.
Middleton P, Shepherd E, Crowther CA. Induction of labour for improving birth outcomes for women at or beyond term. Cochrane Database Syst Rev. 2018;5:Cd004945	Other study design, not RCT. Systematic review (30 RCTs). Overlap with HTA report from 2012 (Wennerholm et al., 2012) and the Cochrane review Gulmezoglu et al., 2012. No further trials identified with gestational age according to PICO.
Mirteimouri M, Pourali L, Najaf Najafi M, Ghaffarian Omid M. Intravaginal administration of isosorbide mononitrate for cervical ripening in prolonged pregnancy: a randomised clinical trial. Journal of obstetrics and gynaecology : the journal of the Institute of Obstetrics and Gynaecology. 2019:1-5	Other research question.
Modrzynska A, Radon-Pokracka M, Plonka M, Adrianowicz B, Wilczynska G, Nowak M, et al. Labor induction at full-term and post-term pregnancies. Folia medica Cracoviensia. 2019;59(4):79-94	Other study design, not RCT. Other gestational age in intervention and comparison group.

Mya KS, Laopaiboon M, Vogel JP, Cecatti JG, Souza JP, Gulmezoglu AM, et al. Management of pregnancy at and beyond 41 completed weeks of gestation in low-risk women: a secondary analysis of two WHO multi-country surveys on maternal and newborn health. Reprod Health. 2017;14(1):141	Other study design, not RCT. Other intervention and comparison groups. No data on pregnancy dating.
A clinical trial of induction of labor versus expectant management in postterm pregnancy. The National Institute of Child Health and Human Development Network of Maternal-Fetal Medicine Units. American journal of obstetrics and gynecology. 1994;170(3):716-23	Other gestational age in one or both comparison groups.
Nippita TA, Trevena JA, Patterson JA, Ford JB, Morris JM, Roberts CL. Inter-hospital variations in labor induction and outcomes for nullipara: an Australian population-based linkage study. Acta Obstet Gynecol Scand. 2016;95(4):411-9	Other study design, not RCT. Other intervention and comparison groups. No data on pregnancy dating.
Ocon L, Hurtado R, Coteron JJ, Zubiria A, Ramirez O, Garcia JA. Prolonged pregnancy: procedure guidelines. Progresos de Obstetricia y Ginecologia. 1997:101-6	Other gestational age in one or both comparison groups.
Pyykönen A, Tapper AM, Gissler M, Haukka J, Petaja J, Lehtonen L. Propensity score method for analyzing the effect of labor induction in prolonged pregnancy. Acta Obstet Gynecol Scand. 2018;97(4):445-53	Other study design, not RCT. Other intervention and comparison groups.
Raviraj P, Shamsa A, Bai J, Gyaneshwar R. An Analysis of the NSW Midwives Data Collection over an 11-Year Period to Determine the Risks to the Mother and the Neonate of Induced Delivery for Non-Obstetric Indication at Term. ISRN Obstet Gynecol. 2013;2013:178415	Other study design, not RCT. Other intervention and comparison groups. No data on pregnancy dating.
Roach VJ, Rogers MS. Pregnancy outcome beyond 41 weeks gestation. International journal of gynaecology and obstetrics: the official organ of the International Federation of Gynaecology and Obstetrics. 1997;59(1):19-24	Other gestational age in one or both comparison groups.
Rosenstein MG, Cheng YW, Snowden JM, Nicholson JM, Caughey AB. Risk of stillbirth and infant death stratified by gestational age. Obstet Gynecol. 2012;120(1):76-82	Other study design, not RCT. Other intervention and comparison groups.
Rydahl E, Eriksen L, Juhl M. Effects of induction of labor prior to post-term in low-risk pregnancies: a systematic review. JBI Database System Rev Implement Rep. 2019;17(2):170-208	Other study design, not RCT. Systematic review. Other intervention and comparison groups.
Rydahl E, Declercq E, Juhl M, Maimburg RD. Routine induction in late-term pregnancies: follow-up of a Danish induction of labour paradigm. BMJ open. 2019;9(12):e032815	Other study design, not RCT. Other intervention and comparison groups.
Sahraoui W, Hajji S, Bibi M, Nouira M, Essaidi H, Khairi H. [Management of pregnancies beyond forty-one week's gestation with an unfavorable cervix]. Journal de gynecologie, obstetrique et biologie de la reproduction. 2005;34(5):454-62. Prise en charge obstetricale des grossesses prolongees au-dela de 41 semaines d'amenorrhee avec un score de Bishop defavorable	Other gestational age in one or both comparison groups.
Sargunam PN, Bak LLM, Tan PC, Vallikkannu N, Noor Azmi MA, Zaidi SN, et al. Induction of labor compared with expectant management in term	Other research question. Other gestational age in intervention and comparison group.

nulliparas with a latent phase of labor of more than 8 hours: a randomized trial. BMC pregnancy and childbirth. 2019;19(1):493	
Schwarz C, Schafers R, Loytved C, Heusser P, Abou-Dakn M, Konig T, et al. Temporal trends in fetal mortality at and beyond term and induction of labor in Germany 2005-2012: data from German routine perinatal monitoring. Arch Gynecol Obstet. 2016;293(2):335-43	Other study design, not RCT. Other intervention and comparison groups. No data on pregnancy dating.
Stock SJ, Ferguson E, Duffy A, Ford I, Chalmers J, Norman JE. Outcomes of elective induction of labour compared with expectant management: population based study. BMJ. 2012;344:e2838	Other study design, not RCT. Other intervention and comparison groups. No data on pregnancy dating.
Suikkari AM, Jalkanen M, Heiskala H, Koskela O. Prolonged pregnancy: induction or observation. Acta Obstetricia et Gynecologica Scandinavica Supplement. 1983:58	Other gestational age in one or both comparison groups.
Tsakiridis I, Mamopoulos A, Athanasiadis A, Dagklis T. Induction of Labor: An Overview of Guidelines. Obstetrical & gynecological survey. 2020;75(1):61-72	Other study design, not RCT. A descriptive review of guidelines for induction of labour
Witter FR, Weitz CM. A randomized trial of induction at 42 weeks gestation versus expectant management for postdates pregnancies. American journal of perinatology. 1987;4(3):206-11	Other gestational age in one or both comparison groups.
Wolff SL, Lorentzen I, Kaltoft AP, Schmidt H, Jeppesen MM, Maimburg RD. Has perinatal outcome improved after introduction of a guideline in favour of routine induction and increased surveillance prior to 42 weeks of gestation?: A cross-sectional population-based registry study. Sex Reprod Healthc. 2016;10:19-24	Other study design, not RCT. Other intervention and comparison groups.
Zenzmaier C, Leitner H, Brezinka C, Oberaigner W, Konig-Bachmann M. Maternal and neonatal outcomes after induction of labor: a population-based study. Arch Gynecol Obstet. 2017;295(5):1175-83	Other study design, not RCT. Other intervention and comparison groups.
Zizzo AR, Kirkegaard I, Pinborg A, Ulbjerg N. Decline in stillbirths and perinatal mortality after implementation of a more aggressive induction policy in post-date pregnancies: a nationwide register study. Acta Obstet Gynecol Scand. 2017;96(7):862-7	Other study design, not RCT. Other intervention and comparison groups.
