# Supplementary material for: Induction of labour at 41 weeks or expectant management until 42 weeks: A systematic review and an individual participant data meta-analysis of randomised trials
Source: PLoS Med. 2020 Dec 8;17(12):e1003436. doi: 10.1371/journal.pmed.1003436 (PMC7723286; doi:10.1371/journal.pmed.1003436)
Supplement: Attachment — Submitted filename: alkmark.pdf [file pmed.1003436.s011.pdf]

This paper presents an individual participant meta–analysis of trials of induction of labour.

I was asked for a statistical report and I interpret that to include all aspects of the design and conduct of the study.

Points of detail

Page 11 Excluding studies published in other languages can lead to bias. I note from page 54 that the authors have listed these and fortunately the only one they found is not a trial. Are they convinced that there are no other studies in other literatures like Spanish or (Brazilian) Portuguese?

Page 13 The term external validity is familiar to me but in this context directness sounds unusual. Unless this is just me I would use external validity later rather than directness.

Page 15 to 17 I am confused about exactly what was done for the two different styles of analysis. The authors state here they used Peto's method to compute odds ratios but then go on to state they used log–binomial models which, as they correctly suggest, lead to risk ratios.

Page 16 Deciding to choose the meta–analysis model based on observed heterogeneity is often recommended but these days it is usually preferred to base it on the scientific situation. See Hedges and Vevea (1998) for an early argument and Rice et al. (2018) for a detailed modern version. To quote the latter:

> Measures of heterogeneity should not be used to determine whether fixed effects analysis is appropriate, but users should instead make this decision by deciding whether fixed effects analysis — or some variant of it — answers a question that is relevant to the scientific situation at hand.

Using terminology from Rice et al. (2018) I would have thought that the fixed effects model as opposed to the common effect model or the random effects model was arguably the most appropriate but obstetrics is not my area of science.

Page 18 The portion of text starting n=600 appears in two places but I think it would be better if it only appeared in the right hand column

Page 21 Just for the record it is of course fine not to do significance tests in Table 2.

Page 24 I think I would have used NNT rather than introducing a new term unless obstetricians usually use NNI. To be fair it is explained in the glossary.

Page 25 I do not understand why some of these are not estimable. I can see that the log–binomial model has problems with Neonatal mortality with only one case but most of the others have cases in both groups.

Page 28 Unless I have completely misunderstood I think significance testing for time from randomisation or onset of delivery is meaningless as these are fixed to be different by design.

Page 35 Using random effects does not account for heterogeneity, it is a different model. Using meta–regression might be said to account for (some of) the heterogeneity.

Page 48 Having stated, wrongly in my view, that heterogeneity would drive the choice between fixed and random effects the authors here use a random effects model with $\tau^2 = 0.0$ which is inconsistent.

Page 52 A bit of what I assume to be Swedish has crept in here. It is easy to guess what is meant but perhaps better to make it consistently English.

There have been attempts to integrate IPD and aggregate meta–analyses. A recent article (Phillippo et al., 2020) provides a new approach and references the earlier attempts. Software for it has been provided too `https://CRAN.R-project.org/package=multinma` I can understand if the authors feel that might be a step too far.

Point of more substance

The authors have not found many trials which fit their criteria. I can understand the reluctance to include other study designs although I might have considered cluster trials. Many trials seem to have been excluded over gestational age. I have not looked at any of them but given the lack of trials in the current review are the authors sure that they could not have included some of these other trials and used gestational age as a moderator? As the authors mention it is difficult to study rare events without truly large samples.

Summary

Despite the length of my comments none of these should pose too many problems.

Michael Dewey

References

L V Hedges and J L Vevea. Fixed– and random–effects models in meta–analysis. *Psychological Methods*, 3:486–504, 1998.

D M Phillippo, S Dias, A E Ades, M Belger, A Brnabic, A Schacht, D Saure, Z Kadziola, and N Welton. Multilevel network meta–regression for population–adjusted treatment comparisons. *Journal of the Royal Statistical Society — Series A*, 183:1189–1210, 2020.

K Rice, J P T Higgins, and T Lumley. A re–evaluation of fixed effect(s) meta–analysis. *Journal of the Royal Statistical Society*, 181:205–227, 2018.
