# Supplementary material for: Induction of labour at 41 weeks or expectant management until 42 weeks: A systematic review and an individual participant data meta-analysis of randomised trials
Source: PLoS Med. 2020 Dec 8;17(12):e1003436. doi: 10.1371/journal.pmed.1003436 (PMC7723286; doi:10.1371/journal.pmed.1003436)
Supplement: Attachment — Submitted filename: REVIEWERS COMMENTS IPDA 20200903 final.docx [file pmed.1003436.s012.docx]

**Reviewers’ comment and authors response**

Manuscript D-20-03510R1 for PLoS Medicine

Requests from the editors:

Please note PLOS' data policy (<https://journals.plos.org/plosmedicine/s/data-availability>) and add a non-author contact for readers interested, in the event of publication, in inquiring about access to study data.

Our data availability statement is: Data cannot be shared publicly directly because use of the data from the individual trials was only approved by the ethical committee for use in this IPDA-MA. Approval for use for other purposes would be needed from the individual trials. Contact information for each study is given in supplement Table S6.

In the title, please replace the semicolon with a colon.
We have replaced the semicolon with a colon as requested.

In the abstract, please quote aggregate demographic details for study participants.

We added the following information in the abstract: “*Baseline characteristics were similar between the groups regarding age, parity, BMI and higher level of education.*”

Please add a new final sentence to the "methods and findings" subsection of your abstract, quoting 2-3 of the study's main limitations.

We added the following information in the abstract: “*A limitation of this IPD-MA was the risk of overestimation of the effect on perinatal mortality due to early stopping of the largest included trial for safety reasons after advice of the Data Safety Monitoring Board. Furthermore, only two RCTs were eligible for the IPD-MA, thus the possibility to assess severe adverse neonatal outcomes with few events was limited.*”

At line 83, please begin the subsection with "In this study, we found that ..." or similar (and again at line 675).

We adapted the text exactly as requested above.

Where available, please quote p values alongside 95% CI throughout the paper.

We quoted p values as requested throughout the manuscript.

Rather than "p<0.0001", please quote exact p values or p<0.001 throughout the ms.

We exchanged p<0.0001 for p<0.001 where applicable throughout the manuscript.

Throughout the ms, please adapt reference call-outs to the following format: "... 16% [4,5]." (i.e., no spaces within the square brackets).

We adapted the reference call-outs throughout the manuscript as requested.

Please use the spelling "fetus" (and derivatives) throughout the article.
We replaced “foetus” with “fetus” in the whole manuscript.

Please abbreviate journal names in your reference list (e.g., "PLoS Med.").

We have corrected the journal names as requested.

Please add a completed checklist for the most appropriate reporting guideline, which we imagine will be PRISMA, as a supplementary file (referred to in the methods section; "See S1_PRISMA_Checklist"). In the checklist, individual items should be referred to by section (e.g., "Methods") and paragraph number rather than by line or page numbers, as the latter generally change in the event of publication.

We included this as a supplementary file and refer to this in the methods section: “*see S1 text PRISMA checklist.”*

Comments from the reviewers:

*** Reviewer #1: Michael Dewey

This paper presents an individual participant meta{analysis of trials of induction of labour.

I was asked for a statistical report and I interpret that to include all aspects of the design and conduct of the study.

**Points of detail**

Page 11 Excluding studies published in other languages can lead to bias. I note from page 54 that the authors have listed these and fortunately the only one they found is not a trial. Are they convinced that there are no other studies in other literatures like Spanish or (Brazilian) Portuguese?

We extended our search to all languages and found no additional trials. In the manuscript we removed the language restrictions:” We *used no limitation for publication year or language. ~~Publications had to be in English, Swedish, Norwegian, Danish or Dutch.).~~*” Supplement S2 Text has been altered with additional information on an extended search without language limitation. Further, the flow chart (Figure 1) has also been altered accordingly with an additional box for the second search.

Page 13 The term external validity is familiar to me but in this context directness sounds unusual. Unless this is just me I would use external validity later rather than directness.
In the GRADE approach (in)directness is used, both terms are interchangeable (*BMJ Evidence based medicine 2018 Feb;23(1):17-19*). We changed directness into external validity.

Page 15 to 17 I am confused about exactly what was done for the two different styles of analysis. The authors state here they used Peto's method to compute odds ratios but then go on to state they used log-binomial models which, as they correctly suggest, lead to risk ratios.

We changed the order in the text in the method section, subheading “synthesis methods and data analysis”, to clarify the description of the methods, and added explanatory text. Hopefully this is better. “*One-step MA was done for those outcomes where individual participant data could be used.*

*For dichotomous outcomes, RR and RD were estimated using generalised linear models with a log- or identity-link and binomial distribution, respectively. A categorical covariable coding for the study was used to permit for within-study associations. For all risk estimates 95% confidence intervals (CI) and p-values were calculated.*

*In case of a zero-event outcome in one arm of one or both trials, Peto odds ratios (OR) were calculated using a two-step approach. In case of zero events in both arms (double zero events) in all but one RCT, no risk estimates or inferential statistics were calculated because double zero events will add zero weight to the analysis. Continuity correction for sparse events was not used.”*

Page 16 Deciding to choose the meta-analysis model based on observed heterogeneity is often recommended but these days it is usually preferred to base it on the scientific situation. See Hedges and Vevea (1998) for an early argument and Rice et al. (2018) for a detailed modern version.

To quote the latter: **Measures of heterogeneity should not be used to determine whether fixed effects analysis is appropriate, but users should instead make this decision by deciding whether fixed effects analysis | or some variant of it | answers a question that is relevant to the scientific situation at hand.** Using terminology from Rice et al. (2018) ***I would have thought that the fixed effects model as opposed to the common effect model or the random effects model was arguably the most appropriate*** but obstetrics is not my area of science.

We had planned to report estimates based on a fixed effects model, though later decided to apply DerSimonian and Lairds method because it is still propagated e.g. in the Cochrane handbook. We have now reported all estimates with adjusted relative risk because they are similar to the estimates calculated with the fixed effects model.

Out of all outcomes, there were only seven in which heterogeneity was observed (see below).

| **Variable** | **I^2^**  **(%)** | **Adjusted relative risk**  **(95% CI)** | **Fixed effect model**  **(95% CI)** | **Random effect model**  **(95% CI)** |
| --- | --- | --- | --- | --- |
| Neonatal infection or sepsis | 52 | 0.83 (0.57; 1.21) | 0.84 (0.58; 1.22) | 0.78 (0.44; 1.41) |
| Apgar score < 7 at 5 minutes of live births | 66 | 0.74 (0.46; 1.19) | 0.75 (0.46; 1.22) | 0.74 (0.32; 1.70) |
| Small for gestational age <3rd percentile | 83 | 0.82 (0.54; 1.27) | 0.84 (0.54; 1.31) | 0.81 (0.27; 2.43) |
| Use of oxytocin | 89 | 1.33 (1.26; 1.40) | 1.32 (1.26; 1.40) | 1.37 (1.15; 1.63) |
| Antibiotics during labour | 62 | 0.99 (0.85; 1.15) | 0.99 (0.85; 1.14) | 1.08 (0.76; 1.53) |
| Postpartum haemorrhage (>1000 ml) | 70 | 0.98 (0.82; 1.17) | 1.02 (0.84; 1.22) | 1.06 (0.74; 1.53) |
| Retained placenta with haemorrhage ≤1000 ml | 63 | 1.07 (0.65; 1.77) | 1.06 (0.63; 1.77) | 1.14 (0.47; 2.76) |

The incidence of most of these outcomes was low, and estimates are based on few events. An exception is oxytocin, which use in clinical practice is different in Sweden and the Netherlands. We have updated the results with adjusted relative risk instead of using random effect model.

In the text addressing heterogeneity, we removed: ~~“~~*~~If I~~^~~2~~^ ~~≥50% fixed-effect RR was calculated.”~~*

Page 18 The portion of text starting n=600 appears in two places but I think it would be better if it only appeared in the right hand column.

Thank you for this suggestion, we removed it from the left box and kept it in the right box.

Page 21 Just for the record it is of course fine not to do significance tests in Table 2.

Thank you for your confirmation.

Page 24 I think I would have used NNT rather than introducing a new term unless obstetricians usually use NNI. To be fair it is explained in the glossary.

We replaced NNI by NNT throughout the manuscript.

Page 25 I do not understand why some of these are not estimable. I can see that the log-binomial model has problems with Neonatal mortality with only one case but most of the others have cases in both groups.

We unintentionally used the word estimable, this should have been ‘estimated’. We have changed the text accordingly and specified the reason for this in the legend of the table: *“NE = Not estimated due to zero events in both arms in one of the trials”*

We decided not to calculate Peto OR for outcomes with double zero in any of the studies. This occurred for nine outcomes. Although the Peto OR method does not require zero-cell corrections, trials with double zero events are given zero weight and will be excluded from the analysis (Bradburn et al., Statistics in Medicine, 2007. 26 (1): 53-77). Thus, for these outcomes we refer to the original SWEPIS and INDEX publications, respectively. Continuity correction for sparse events was not used. Thus, for these outcomes we refer to the original SWEPIS and INDEX publications, respectively.

Page 28 Unless I have completely misunderstood I think significance testing for time from randomisation or onset of delivery is meaningless as these are fixed to be different by design.

While this may appear illogical, there is in fact reason to do this. In such trials the allocated treatment cannot always be implemented in practice. For example, if compelling clinical reasons require immediate delivery but the patient was in the expectant management group. It is therefore useful to help the reader understand if observed effects are indeed in-line with the intended protocol. In addition, reviewer #3 asked for the p-value for gestational age at delivery.

Page 35 Using random effects does not account for heterogeneity, it is a different model. Using meta regression might be said to account for (some of) the heterogeneity.

We adapted the text into: *“The heterogeneity between trials for most outcomes was low. Only for one important outcome (Apgar score) heterogeneity was considerable.”* We removed: ~~“~~*~~which was accounted for by using random effect relative risks.”~~*
Given that the most important outcomes are rare, meta-regression is not feasible.

Page 48 Having stated, wrongly in my view, that heterogeneity would drive the choice between fixed and random effects the authors here use a random effects model with τ2 = 0:0 which is inconsistent.

If no heterogeneity is observed, the outcomes using the DerSimonian and Laird method is not different from the fixed effects outcomes. The random effects method however, is now no longer used and the text has been changed accordingly. We rephrased the text under the subheading Meta-analysis on aggregate data: “*If IPD was not available, MA was performed on aggregate data from eligible trials. In this situation relative risks were calculated using a Mantel-Haenszel fixed effect model, or Peto ORs were calculated as appropriate.”*

Page 52 A bit of what I assume to be Swedish has crept in here. It is easy to guess what is meant but perhaps better to make it consistently English.

Apologies, we have overlooked this, we altered the text into what we believe is proper English.

*“Search, query, results, limitation and publication date”*

There have been attempts to integrate IPD and aggregate meta-analyses. A recent article (Phillippo et al., 2020) provides a new approach and references the earlier attempts. Software for it has been provided too <https://CRAN>. R-project.org/package=multinma I can understand if the authors feel that might be a step too far.

Thank you for this suggestion. We will consider this for a next IPD-MA. In this IPD-MA we have prespecified in our PROSPERO protocol the strategy of data synthesis and followed this accordingly in this manuscript. No changes were made to the revised manuscript since indeed we feel for now this is also a step too far

**Point of more substance**

The authors have not found many trials which fit their criteria. I can understand the reluctance to include other study designs although I might have considered cluster trials. Many trials seem to have been excluded over gestational age. I have not looked at any of them but given the lack of trials in the current review are the authors sure that they could not have included some of these other trials and used gestational age as a moderator? As the authors mention it is difficult to study rare events without truly large samples.

Indeed, we agree that this is a key point for consideration. At the start of the IPD MA process we have discussed extensively in our research team if we should include trials with other time frames of comparison. We decided not to do this because we aimed for a strict comparison between induction at 41 and 42 weeks. Expectant management beyond 42 weeks is abandoned as regular policy since many years because of a known substantial increase of perinatal mortality after 42 weeks. Furthermore, these other studies were done between 15-40 years ago meaning they are not representative anymore for today’s pregnancy surveillance and/or routine method of estimated date of delivery (nowadays early ultrasound measurement). Including trials with induction before or after 41 weeks and an expectant management policy far beyond 42 weeks would have caused methodological problems. As moderation analysis is performed using individual or contextual factors at baseline that may affect the relationship between intervention and outcome, we assumed that gestational age was not suitable as potential moderator.

We found no clustered randomised trials on the subject.

We added in the discussion a sentence addressing this issue: *“Including IPD from trials with induction before or after 41 weeks and an expectant management policy beyond 42 weeks would have caused methodological problems such as selection bias.”*

**Summary**

Despite the length of my comments none of these should pose too many

problems.

Michael Dewey

References

L V Hedges and J L Vevea. Fixed- and randome_ects models in meta{

analysis. Psychological Methods, 3:486{504, 1998.

D M Phillippo, S Dias, A E Ades, M Belger, A Brnabic, A Schacht,

D Saure, Z Kadziola, and N Welton. Multilevel network meta{regression

for population{adjusted treatment comparisons. Journal of the Royal Sta-

tistical Society | Series A, 183:1189{1210, 2020.

K Rice, J P T Higgins, and T Lumley. A re{evaluation of _xed e_ect(s)

meta{analysis. Journal of the Royal Statistical Society, 181:205{227, 2018.

*** Reviewer #2:
Review of manuscript D-20-03510R1 for PLOS Medicine
Thank you for the opportunity to review the manuscript "Induction of labour at 41 weeks or expecting management until 42 weeks; an individual participant data meta-analysis of randomized trials", currently under consideration for publication in PLOS Medicine.
The aim of the study was to evaluate the effect of IOL at 41 weeks versus expectant management until 42 weeks on important and especially rare perinatal and maternal outcomes. We also aimed to assess whether treatment effects differed in subgroups.

Overall, the paper examine an important and complex topic and the paper is well-written. However, there are some major concerns when it comes to the methodology, data analysis, interpretation of the results, and the scientific ambitions of the paper.
I have elaborated my main concerns below:

**Introduction:**
In the introduction the body of evidence on the current topic is expected to be introduced for the reader. In the present introduction the two studies from the authors and the Cochrane meta-analysis are the overall main evidence. However, there are other randomized studies, meta-analysis (41 vs 42 weeks), and quasi-experimental studies but they are not presented and discussed - this is a major limitation of the study and need to be improved making a balanced introduction on the body of evidence to the reader.

Thank you for this suggestion. We adjusted the Introduction: “*A recently published systematic review including two RCTs, two quasi-experimental trials and three retrospective cohort studies compared IOL at 41+1 to 41+6 weeks with expectant management until 42+0 to 42+6 weeks. IOL at 41+1 to 41+6 weeks was found to be associated with an increased risk of caesarean delivery and pH < 7.10, but it lacked power to estimate the risk of perinatal mortality. However, the timeframe in this systematic review allows comparison beyond 41 weeks with expectant management until 43 weeks, which is not comparable with our timeframe. We chose an upper limit of 42 weeks because continuing pregnancy after 42 weeks is no longer regular policy since many years due to its association with increased perinatal mortality. This is reflected in many national and international guidelines.”* We only included RCT’s because we aimed for a straight comparison of two management strategies with the lowest risk of selection bias.

In the last part of the introduction the aim of the study is presented - the authors want to study "important" and rare outcomes - but there are important outcomes not included in the analysis provided by the authors - I suggest the authors are more objective in their aim and reflect on which words they use.

The sentence has now been rephrased: “*The objective of the IPD-MA was to evaluate the effect of IOL at 41 weeks versus expectant management until 42 weeks on perinatal and maternal outcomes, with a focus on rare adverse perinatal outcomes.*”

We also rephrased this in the method section under subheading specific objectives.

**Method:**
In general, information on the limitations on the two studies included in the analysis are limited. This is a limitation as it has impact on how to treat data and make conclusions. Especially, the Swedish trial have several limitations (stopped early, violation of protocol etc) The authors do know of these limitations (also stated in the many responses to the published study) and should ensure transparency to these limitations in the present paper. A meta-analysis is not getting better that the study included in the analysis.

We added the early stopping of SWEPIS in the Abstract “*A limitation of this IPD-MA was the risk of overestimation of the effect on perinatal mortality due to early stopping of the largest included trial for safety reasons after advice of the Data Safety Monitoring Board. Furthermore, only two RCTs were eligible for the IPD-MA, thus the possibility to assess severe adverse neonatal outcomes with few events was limited.*”, in the Results section we already stated that: “*It had some problems regarding study limitations for the outcome perinatal mortality.*” And we added in the Conclusion “*The magnitude of risk reduction of perinatal mortality could be affected by the early stopping of the SWEPIS trial.”*

Furthermore, we would like to stress that there was no limitation due to protocol violation in the SWEPIS trial. A preliminary version of the protocol was published by Elden et al. (April 2015), the final study protocol, approved by the ethical committee, was set in early 2016 (March 11, 2016) well before the start of the SWEPIS trial in May 2016. The final study protocol did not include routine ultrasound at 41 weeks, pregnancy checks were allowed to be performed according to local protocol, thus increasing external validity. Finally, we agree with the remarks of reviewer 3 on this: *The authors have applied the gold-standard of IPD-MA to attempt to answer this question, and have used this to advantage in pre-specifying a composite adverse perinatal outcome that truly reflects substantial morbidity, unlike the "softer" adverse outcomes such as Apgar at 5 minutes <7 that are often used in individual RCTs.”*

**Outcome:**
This study mainly use data from two previously published RCTs - one study from the Netherlands and a ¼ of a RCT study from Sweden. The main outcome is "rare perinatal and maternal outcomes" even though rare outcomes are very hard to examine in RCT (that's why we normally use other and more robust methods when studying mortality/rare outcomes)
Rare outcomes has a nature of a fluctuate pattern in its distribution and therefor the possibility of stopping the trial earlier than expected is high and it follow with an overestimation of the studied effect - **the authors acknowledge the risk of overestimation from the early stopped trial but do not take it into considerations when forming their conclusion -this is a major shortcoming of this present study.**

We added more information about this aspect to the Abstract, and also in the Conclusion (please see above). We recognise that the early stopping of the SWEPIS trial may affect the estimated risk, particular for an outcome with low occurrence. Some experts, but not all, have stated that trials stopped early tend to overestimate treatment effects. This however, is a discussion mainly in the context of trials stopped for benefit. The SWEPIS trial was, however, stopped for safety. Stopping a trial for safety is another matter than stopping for benefit (Goodman, Ann Intern Med. 2007 Jun 19;146(12):882-7; Goodman, J Clin Epidemiol. 2008 Jan;61(1):95-6; Berry et al., JAMA. 2010 Jul 14;304(2):156; author reply 158-9; Goodman et al., JAMA. 2010 Jul 14;304(2):157; author reply 158-9; Wang et al., Clin trials. 2016;13(6):621-31; Pocock, JAMA.2005 294 (17)2228-2230; Pocock et al., J Am College of Cardiology. 2015 66(25)2886-2898).

**We do also know that the mortality in the swepis trial was higher than the mortality is in Sweden in general - pointing towards an overestimation of the effect and a fluctuate pattern.**
Another important issued with the outcome is when studying mortality, we need to know more of the cases before we can calculate a number needed to treat (as the authors do) in other **words if we conclude we are able to prevent some cases we need the case fatality not the total mortality as presented by the authors.** I have had a look into stillbirth and perinatal deaths from week 41 and onwards in my country and found that some of the cases where not preventable - more information are therefor needed before number needed to treat is calculated and ***the conclusion needs to be moderated if we are not dealing with the case fatality (this is common knowledge in public health and mortality research but not always in the obstetric field)***

Concerning the mortality rate in SWEPIS and in the Swedish background population the differences for stillbirth and perinatal mortality is difficult to interpret due to the rarity of these events. A significantly increased risk of stillbirth, when late term pregnancies continue to 42 weeks compared to delivery at 41 weeks was identified in a recent systematic review and meta-analysis of cohort studies (Muglu et al., PLoS Med. 2019 Jul 2;16(7):e1002838). Further, the way data on stillbirths and neonatal mortality were obtained may not be quite comparable between the study population and the background population. The data on stillbirths from the background population represent register data from the Pregnancy Register (Stephansson et al., Acta Obstet Gynecol Scand. 2018 Apr;97(4):466-476) from almost all clinics in Sweden during the study period, yet, it has not been validated against the medical records as in SWEPIS. The background population includes neonatal deaths from the Swedish Neonatal Quality Register (Norman et al., Acta Paediatr.2019 Aug;108(8):1411-1418), but not any vital statistics from Statistics Sweden (<http://www.scb.se/en>), which we used in SWEPIS to obtain data on neonatal mortality <28 days. Finally, there are unexplained differences in stillbirth rates in different regions in Sweden (https://stillbirth.se/wp-content/uploads/2019/01/Socialstyrelsens-rapport). Sweden has a rather high stillbirth rate (3.7/1000 in 2016) compared to other Nordic and many European countries and there has been no decline in stillbirth rates during the latest years in Sweden (https://stillbirth.se/wp-content/uploads/2019/01/Socialstyrelsens-rapport). We agree that due to few events the magnitude of the reduced stillbirth/neonatal mortality in late term pregnancies is still imprecise.

In our manuscript we described the perinatal deaths “*The only perinatal death in the IOL group was a stillbirth that occurred one day after randomisation but before IOL. In the expectant management group seven of the eight deaths were stillbirths and one baby died after birth due to hypoxic ischemic encephalopathy. In post mortem examination of one of the stillbirths a non-lethal cardiovascular malformation was found, two of the stillbirths were SGA (both between the 5th and 10th centiles). In the other six cases no possible explanation was found*.” So we did not find direct causes for the mortality. We think this is enough detailed information and we cannot give more details on case level due to privacy reasons.

Before we are able to make a meta-analysis it is important to access if the outcome cases fulfil the inclusion criteria - this is questionable for the Swedish trial as the UL assessment at 41+0 was not conducted for many of the cases. The authors need to provide information on how many of the cases that fulfilled the inclusion criteria (UL assessment) at 41+0. There is some very small fetuses (SGA) which in normal practice would have been found if they have had an UL scan in 41+0 - meaning risk pregnancies were included in the trial (SGA children have 5-6 times higher mortality) violating the inclusion criteria.

As explained above, there was no protocol violation in the SWEPIS study, as all cases fulfilled the inclusion criteria. Ultrasound at study entry was not mandatory and we added*: “it was not an inclusion criterion.”* in the discussion under strength and limitations where we reflect on this matter. Unfortunately, we have no information on ultrasounds performed at study entry outside the Stockholm region.

**Exposure:**
What is the exposure in expectant management regime - this has not been specified - as this is important as it is the exposure - it is not enough to make a general statement as provided in the present manuscript - if the authors do not have a clear definition (and compliance) of the regime of expectant management in the two studies this needs to be clearly stated (see line 197) and conclusion may be downgraded if a clear statement of expectant management is not possible to describe. Description of expectant management may include different kinds of surveillance, number of antenatal visits etc)

We have described the regimes of expectant management in Table 1. (INDEX: “*Fetal surveillance in the EM group was performed according to local protocols and could include cardiotocography and/or an ultrasound assessment of amniotic fluid*”; SWEPIS: “*Fetal surveillance in the EM group was performed according to local protocols and typically included antenatal visits with auscultation of the fetal heart rate*”. We performed a pragmatic trial thus reflecting clinical practice in both countries, which increases the external validity.

To clarify, we have added in the text: “*Fetal surveillance, in the two trials included in the IPD-MA, in expectant management group was performed according to local protocol (Table 1).”*

We also added in the discussion under strength and limitations: “*This could also be considered as a strength, rather than a limitation, because it increases external validity of these pragmatic trials.”*

**Risk of bias:**
In line 240 it is stated that risk of bias was assessed independently by some of the authors - the results of these scores are found in table 3 - both the Swedish trial and the trial from The Netherland is almost scores with low risk of bias in all categories despite of serious limitations especially in the Swedish trial affecting the level of bias in more than one category - more transparency is needed on the scoring process and maybe a more independently scoring process is needed to gain a more accurate and valid result.

The presented low risk of bias is in agreement with the assessments in the Cochrane 2020 publication (Middleton et al., Cochrane Database of Systematic Reviews 2020, Issue 7. Art. No.: CD004945. DOI: 10.1002/14651858.CD004945.pub5) We have added as a possible limitation in the discussion: *“Risk of bias assessed by the authors themselves may be considered as not independent, however, the low risk of bias is in agreement with the assessment of the independent reviewers in the Cochrane 2020 updated review of Middleton et al.”*

**Please also remember the low participation rate in both studies and how it affect the validity of the study**

Indeed, a substantial proportion of eligible women declined randomisation in both RCTs, however there were no cases lost to follow up and in the trials the most important background characteristics are similar to the population of women declining participation (INDEX) or the comparable general population (SWEPIS).

**Results:**
It is well known that using composite outcome measures for rare outcomes in RCT is often inadequate in terms of extrapolation to clinical setting (but sufficient in relation to statistics one could say greater precision but with greater uncertainty) and the results gained more often apply to an individual measure rather to the composite. A high proportion of trials using composite outcome measures do not report significant results for the mortality component but for the composite outcome measure and further, very few of the trials with composite outcomes finds significant results for the mortality component but not for the composite outcome measure - this illustrate why composite outcome measure are very difficult to use when making clinical conclusions. The authors do not discuss this well-known challenge and do neither take it into account when forming their conclusion. More information on this can be found in the publication by Freemantle or Cordoba or McKenna among others if the authors are not familiar with this challenge.
Due to the low incidence of severe adverse perinatal outcomes, a composite outcome was used in both trials and also in this IPD-MA. This outcome gives relevant information on the short term health situation of the neonate. Perinatal mortality was a component of the composite, but also reported separately in order to provide insight in the main outcome of interest of many caregivers.

In the SWEPIS trial the composite perinatal outcome was not significant, but perinatal mortality was. In the INDEX trial the composite perinatal outcome was significant, but perinatal mortality was not. An IPD-MA gives the opportunity to enlarge the study population resulting in more precise risk estimations, also of rare outcomes.

One may criticise the choice of the components of the composite outcome, but we have tried to choose a composite of the most relevant severe adverse perinatal outcomes. Therefore, we altered Apgar <7 to Apgar < 4, because these neonates are more at risk of cerebral palsy. We give some more explanation in the Methods section: “*as an Apgar score <4 at five minutes “can be considered as a non-specific sign of illness, which* *may be one of the first indications of encephalopathy*” according to the AAP and ACOG (Committee Opinion No. 644, 2015). See also the favourable comments on this from reviewer # 3: *“The authors have applied the gold-standard of IPD-MA to attempt to answer this question, and have used this to advantage in pre-specifying a composite adverse perinatal outcome that truly reflects substantial morbidity, unlike the "softer" adverse outcomes such as Apgar at 5 minutes <7 that are often used in individual RCTs.”*

In line 435, AAR would be a better measure to calculate - in this case it would be 0.6% - please include this number to gain transparency to the effect found.

We chose not to add the ARR, because this is directly derivable from the incidence as reported: IOL 0.4% [10/2281] versus 1.0% [23/2280] in EM. We have specifically also reported risk differences/10,000 (e.g. -57/10,000 [95% CI -106/10,000 to -8/10,000]) to provide the reader with absolute risks.

In table 4, why is the Apgar <4 at 5 minutes (significant) used as a subcomponent in the primary composite outcome measure - why not Apgar < 7 at 5 minuts (not significant) - what is the result if Apgar <7 is used instead of Apgar < 4. Conventionally we use Apgar less than 7.

As mentioned above we included only the most relevant severe adverse perinatal outcomes in the primary outcome. In Table 4 with perinatal outcomes we also show the primary outcome including Apgar 5’<7 instead of <4. We added some more information in the Methods section with a reference to the ACOG publication on the use of Apgar score in which Apgar score between 4 and 6 is defined “moderately abnormal” and <4 as “low”. An Apgar score of 0–3 at 5 minutes or more can be considered as “*a nonspecific sign of illness, which* *may be one of the first indications of encephalopathy*”. It is also stated that “*Although the (Apgar) score is used widely in outcome studies, its inappropriate use has led to an erroneous definition of asphyxia.*” (Committee Opinion No. 644, 2015).

Please also provide the distribution in the two study groups for Apgar < 4 and 7

This is reported in the manuscript (Table 4) and we provide the distribution in the two trials in the Supplementary table S2.

In table 4, it is also illustrated that most of the death children are from the swepis trial meaning that it is mainly the swepis study (or a ¼ of a study with all it´s limitations) providing the main result for this study. As mentioned before there were some violation to the protocol meaning that most of the women did not have an ultrasound examination upon inclusion in 41+0 and detection of very small fetus was lacking meaning that high risk pregnancies were allocated to expecting management even though the inclusion criteria was low risk singleton pregnancies (we did experience the same in the term breech trial which have had devastating influence on women health and health care professionals expertise)

As we explained above, there was no protocol violation. None of the perinatal deaths had a birth weight below the 5^th^ percentile.

Table 4, the variable NICU > or equal to 4 days need further explanation - why the cutpoint of 4 days? Do the authors have indication for being admitted to NICU

The outcome admission to neonatal care ≥4 days was prespecified in the protocol, and was chosen after consultation with neonatologist to identify real sick neonates. We rephrased the text in the method section to clarify: *“admission to neonatal care (medium care -excluding observation only for protocol- or intensive care unit), admission to neonatal care ≥ 4 days mimicking more intensified neonatal care for sick infants in need for longer treatment and/or more extensive observation. (NICU admission as such could not be used as a variable because of different admission criteria in Sweden and the Netherlands)”*. NICU in Sweden and NICU in the Netherlands are not comparable regarding admission criteria therefore we could not only use the outcome neonatal care admittance. In Swedish NICU’s neonates can be admitted for mild problems or preventive antibiotic treatment until lab results are ready. In the Dutch NICU’s there are strict criteria for admittance allowing only very sick neonates who are in need of life support and/or intensive treatment like intubation. Therefore, we had to develop a new outcome that mimics admission of sick neonates and we decided to apply: “admittance to neonatal care unit ≥ 4 days” because most neonates with minor problems will be discharged within 4 days. The indications for admittance of the 4 days or more are available and, we could provide this information in a supplementary table if that is desired. We also rephrased the text in the discussion under the subheading “Strengths and limitations” on this issue: “*We changed from Apgar <7 to Apgar <4 at five minutes and replaced NICU admission by clearly diagnosed severe neonatal illness and severe complications because NICU admission criteria in Sweden and The Netherlands are not comparable.”*

Table 5. is only 3 days difference between induction of labour and expecting management - this need to be highlighted - as this may tell us that there is minor difference in gestational length in the two groups.

Thank you for the remark, we added this information: “*The median gestational age in the IOL group was 288 (IQR 287; 289) days and 291 (IQR 289;293) in the expectant management group,* which corresponds with 41+1 versus *41+4 weeks, a difference of three days.”*

Table 5, I find a very low c section rate of 10% in both groups - this need to be mention together with the statement of no difference in c-section - as the low rate may leave no potential to study this outcome effectively.

We have added in the discussion: “*The low rate of caesarean delivery reflects the low risk population of this IPD-MA*.” The difference between groups had a narrow 95% CI (RD -0.2%; 95% CI -2.0% to 1.6%, and the incidence of caesarean delivery rate for nulliparous women (18.0% vs 17.9%) is comparable with the ARRIVE trial (18.6% vs 22.2%) (Grobman et al., N Engl J Med. 2018;379(23):2278-9), also we have included multiparous women without previous caesarean delivery, therefore a low caesarean delivery rate could be expected*.*
We also added the RR, 95% CI and p-value in the result section under the subheading Subgroup analysis*: “The rate of caesarean delivery in nulliparous women was 18.0% (219/1,219) in the IOL group and 17.9% (226/1,264) in the expectant management group (RR 1.01 [95% CI 0.85 to 1.19], p-value 0.87). Corresponding rates for multiparous women were 2.0% (21/1,062) and 1.9% (19/1,016) (RR 1.12 [95% CI 0.36 to 3.56], p-value 0.95).”*

Table 5, the most common side-effect of the intervention studied (induction of labour) is hyperstimulation (proxy variable use of medication to slow down contractions) - this clinical relevant information is not included - do the authors have some information on hyperstimulation - if not this need to be stated as a limitation as it is a common side-effect of the intervention studied.
In the SWEPIS trial ICD10 codes are used, but unfortunately there is no ICD10 code for hyperstimulation, The INDEX trial reported the use of tocolytics, but the SWEPIS trial didn’t record tocolytics, therefore we could not report on this in the IPD-MA. However, the main risk of hyperstimulation is caesarean delivery and perinatal asphyxia, and none of those outcomes were increased in the IOL group. We added this information after the caesarean delivery results in the result section under subheading “Delivery outcomes”: *“Uterine hyperstimulation was not registered as such but there was no increase in caesarean delivery after IOL compared to expectant management and there was no difference between the groups with respect to the indication for caesarean delivery.”*

Table 5, meconium stained amniotic fluid (MSAF), 380 cases versus 52 cases - guess there is something wrong with the numbers - is 380 meant to be 38? - this outcome is significant - and this is expected as we will expect more MSAF with longer pregnancy - a physiological explanation - the outcome we worry about is aspiration of MSAF - please balance this (also in your conclusion)

Thank you for this remark, we have overlooked this. We corrected the number. We also added MAS in the results section under subheading “Delivery outcomes: “*The rate of MAS in the IOL group was 0.1% (2/2280) and 0.2% (5/2273) in the expectant management group, RR 0.42 (0.10 to 1.86), p-value 0.25, RD -13/10,000 (-36/10,000 to 10/10,000), I^2^ 0%*.”

Figure 4, interaction is found for parity - this is quite normal. When interaction is found for parity all results from this study needs to be calculated separate for nulliparous and multiparous women as the effect is dependent by parity. The total calculation is biased and is not valid to form conclusions from.
We tested interaction between parity and the primary outcome and caesarean delivery, and intended to perform it for perinatal mortality but this could not be done due to few events. That we found interaction between parity and the primary outcome does not mean that there is interaction with all other outcomes. For the reviewers and readers interest we performed a new interaction analysis on all other outcomes and we only found interaction for use of oxytocin. Therefore, we will not present all outcomes according to parity in our manuscript.

In the methods section under the subheading “IPD- MA” we added: *“In case of significant interaction for any of these outcomes additional analysis was performed on all outcomes for this subgroup.”*

We also added text in the results section under the subheading “Subgroup analysis: *“For other outcomes there were no significant interaction effects for parity, except for use of oxytocin. This was used more frequently in nulliparous women, 76% in the IOL group versus 65% in the expectant management group, corresponding rate for the multiparous women were 49% versus 26%, p-value for interaction <0.001 (S5 table).”*

If desired, we could provide four supplementary tables with outcome data stratified for parity.

Overall, the authors do not have a high internal validity of their study and therefore they need to discuss the limitations of each study and the merged study results better and how the limitations may affect the conclusion.

In addition to statements made above regarding this issue we also rephrased a paragraph in the result section under the subheading “Study quality including risk of bias assessment”: “*In addition, the internal validity of the estimated risk for perinatal mortality can be affected by the early termination of the trial following advice from its Data and Safety Monitoring Board. The board recommended stopping for safety after an increased risk for perinatal mortality was observed at a planned interim analysis. Early termination of RCTs could be associated with overestimating of treatment effect, especially for events with low occurrence. There were no or minor problems regarding study limitation for the other outcomes. Regarding precision, the study had lower power than planned for the composite outcome and perinatal mortality due to the early stopping.”*

We also altered the text in the discussion under the subheading Strengths and limitations: *“The SWEPIS trial was stopped early due to safety reasons. Therefore, the magnitude of the risk on perinatal death could be affected and may be overestimated also considering the difference in perinatal mortality rate between the SWEPIS and INDEX trial”.*

At the moment the conclusion is not supported by the data provided in this paper. A clinical conclusion based on the data from the two studies may be vague as there is several limitations which do not allow for a clear conclusion neither recommendation for clinical practice.

We are surprised that the reviewer concluded that the conclusion is not supported by the data. We do not see how the risk estimates with the 95% confidence intervals could allow for another conclusion. Regarding the suggested limitations, we feel that we have discussed most of them adequately in our previous replies. Although the magnitude of the effect on perinatal mortality could be affected due to the early stopping of one trial, the point estimate goes in the same direction as in the Cochrane review on induction of labour at or beyond 37 weeks, which included more trials (Middleton et al., Cochrane Database of Systematic Reviews 2020, Issue 7. Art. No.: CD004945. DOI: 10.1002/14651858.CD004945.pub5). Although being a subanalysis, an important and clear outcome of our study is that the effect we have found especially applies to nulliparous women, for multiparous women the effect is still unclear. In our opinion this could easily be used and should be used in clinical practice by explaining these results to pregnant women so they can make an informed choice for one of the two strategies.

In the present form, the paper is more a position statement paper rather than a scientific paper
There is a remarkable lack of balanced and relevant discussion of the findings - this is a shame as I expect the authors have had a huge work with data cleaning.  
We politely disagree with this remark, we did our uttermost best to provide a scientific reliable paper in which we adhered to the previously published protocol and reported and analysed according to the existing guidelines. In the Discussion section we answered the research question and attempted to put it into perspective. We hope that our previous replies have also changed the reviewer’s opinion on this remark.

*** Reviewer #3:

This is a well-conducted and well-written piece of work on a topic of considerable importance, namely at what gestation is induction of labour (one of the most common obstetric procedures) in a post-dates pregnancy of demonstrable benefit regarding major adverse perinatal outcomes, and are there maternal adverse outcomes either caused or avoided with IOL? The authors have applied the gold-standard of IPD-MA to attempt to answer this question, and have used this to advantage in pre-specifying a composite adverse perinatal outcome that truly reflects substantial morbidity, unlike the "softer" adverse outcomes such as Apgar at 5 minutes <7 that are often used in individual RCTs. The authors show evidence that, at least for nulliparous women, IOL at 41 weeks leads to better perinatal outcome without increases in obstetric procedures or adverse maternal outcomes (and in the case of hypertensive disorders of pregnancy, a decrease in the IOL group) - and with numbers needed to
induce. These findings are of immediate applicability to counselling women regarding IOL in high-income countries/those with comparable maternity care systems.
Thank you for this positive judgement.

I do however suggest minor revisions as detailed below:
1) Abstract, methods and findings: "Perinatal deaths occurred in one (0.0%)..." - suggest change this to "Perinatal deaths occurred in one (<0.1%)....". I realise that with the numbers involved the rounding down makes 0%, however given that there was in fact one perinatal death, it is less confusing to the reader to say <0.1% rather than 0.0%.

We agree with this remark and changed the manuscript accordingly.

2) Author summary (why was this study done?). The first point notes that in observational studies IOL has been associated with increased risks. However, IOL RCTs for a variety of reasons in a variety of settings do not usually suggest these risks. Therefore suggest to balance the first point by "In observational studies, although not usually in interventional studies, IOL has been associated with....(etc)."

We agree and adapted the text accordingly

In point 3 "According to a recent meta-analysis..." suggest minor change for clarity "According to a recent meta-analysis on aggregate data from randomised controlled trials (RCTs), perinatal mortality was lower after induction of labour at or beyond term compared with a policy of expectant management. However, the upper limit of gestational age for expectant management was not taken into account in this meta-analysis".

We agree and adapted the text accordingly.

3) Author summary (what do these findings mean?). The second point needs to be better expressed, as surely women already have the possibility to choose the timing of their post-dates IOL? Maybe "Women can then make an informed choice regarding IOL at 41 weeks versus awaiting spontaneous onset of labour until 42 weeks."

The point is taken and we adapted the text accordingly.

4) Introduction, first sentence (line 132/133) suggest minor wording change "When to induce labour in overdue or post-term pregnancy has been under debate for many years".

We agree and adapted the text: “*When to induce labour in overdue pregnancies has been under debate since many years.”* We also added “*with a steeper increase after 42 weeks.”* in the end of the next sentence.

5) Introduction, line 146 - minor typo in IOL (the L is not capitalised).

Thank you, we have corrected the typo.

6) Introduction, line 147 - replace "Yet" with "However".

We changed the word.

7) Introduction, line 156-159 when talking about the recent Cochrane review update, needs a little rewording for clarity. I think what is being stated here is that you had already done your IPD-MA analysis and were writing up results when the Cochrane update was published? If so, suggest (or similar) "After results of the current study were already finalised, an update of this Cochrane review was published in which the main conclusion was similar to the previous version

(9). Although subgroup analysis was performed for parity, this was not done for the 41-42 weeks' comparison in the Cochrane review".

We have now adapted the text.

8) Introduction line 162 and throughout - fetal and fetus not "foetal" or "foetus". Both in American English and UK English fetus has no "o".

The spelling has been changed throughout the manuscript.

9) Methods, lines 413, 415-16 and 421 - I am not sure what is meant by "directness". I suspect from the context it means external validity/applicability to settings outside that of the trial itself - however please clarify.

As stated above in our response to reviewer 1: In the GRADE approach (in)directness is used, both terms are interchangeable (BMJ Evidence based medicine 2018 Feb;23(1):17-19). We changed directness into external validity.

10) Result, table 5 - ?p value missing for gestational age at delivery (days)

We added the p-value for gestational age at delivery in table 5.

11) Discussion, lines 597-99: suggest minor rewording for clarity - "In the post hoc subgroup analysis on fetal sex we found that boys rather than girls might benefit from IOL regarding the composite severe adverse perinatal outcome. However, perinatal mortality did not differ by fetal sex, occurring in 5 boys and 4 girls in our IPD-MA."

We have now reworded the text accordingly.

12) Discussion line 626 "advise" rather than "advice".
We have corrected the typo.

13) Discussion line 658 - "shown" rather than "showed".

We have corrected the tense of the word.

14) Discussion, strengths and limitations: The additional limitation of external validity should be touched upon. These data are likely to be highly applicable to high-income countries, particularly those with comparable maternity care systems to The Netherlands and Sweden (i.e. most of Europe, Australia/New Zealand, Canada). They are less likely to be applicable to low or middle-income countries.

Thank you for this suggestion, the text has been rephrased: “*In addition, the results of this IPD-MA are likely to be applicable to high income countries due to the equivalence in healthcare status in the included trials but less applicable to low or middle income countries.*”

15) Please double-check referencing. Some journal names are missing appropriate capitals e.g. reference 20 should be JAMA not Jama.
We have checked and corrected this were applicable.
