# Supplementary material for: Induction of labour at 41 weeks or expectant management until 42 weeks: A systematic review and an individual participant data meta-analysis of randomised trials
Source: PLoS Med. 2020 Dec 8;17(12):e1003436. doi: 10.1371/journal.pmed.1003436 (PMC7723286; doi:10.1371/journal.pmed.1003436)
Supplement: Attachment — Submitted filename: Review of the Manuscript nr 2 af metaanalyse med swepisD.docx [file pmed.1003436.s014.docx]

Review of the Manuscript D-20-03510 for Plos Medicine

Thank you for the opportunity to review the revised revision of Manuscript D-20-03510

Overall, the authors have not addressed the comments and suggestions or provided a relevant argumentation for not addressing the limitations of the paper. In the present form the paper is more a position paper than a scientific paper. Please find my elaboration below

I still do not understand why the authors do not include all relevant body of evidence in their introduction. The authors will not include the meta-analysis published by Rydahl et al. because they also have measures on pregnancies extending 42+0. But the authors includes the Cochrane review with several studies including Hannahs trial (which is making a huge contribution to the final result of the meta-analysis) but quite a few of the studies in the Cochrane review examine pregnancies extending beyong 42+0.

Further, another recent national Danish study by Rydahl (Denmark have a comparable setting to Sweden) examined a new national regime of earlier induction of labour in week 41 found no benefit in mortality using a robust method (interrupted time-series and Poisson regression) when studying rare outcomes (which is quite challenging to study in RCT). You have also experienced this core issue in the SWEPIS trial as you had to stop the trial earlier and ending up with overestimation of mortality in your study (your national Swedish numbers of mortality are lower compared to what you found in your trial)

Even though your are aware of this overestimation which is quite logic when you stop trials early on rare outcomes (due to fluctuate pattern) and you have also provided a sentence about it in your manuscript (elaboration of the fact that your Swedish national number are lower would have be admirable to include in the paper). Further, you forget this fact when making the study conclusion. You do not have valid data to make the conclusion stated in the manuscript – you would need to make a less conclusive conclusion.

One can start a discussion on which studies stopped early that may be affected by overestimation but in this case we have numbers showing that the Swepis trial have overestimated the mortality as we can compare with the national data. The authors need to move forward and not only discuss it but also relate to the actual numbers – this is not done in the study and the authors hold back important information to the readers.

The author reply in their review comments “, in the Results section we already stated that: “It had some problems regarding study limitations for the outcome perinatal mortality.” it is good that the authors stated this in the results section. However, in a scientific paper one also need to discuss the limitation with transparency and clear out how these limitations influence the results. In this case, the limitations (including participants in the study without making sure they are low-risk (to be fair those in the Stockholm center – a part of the trial had a scan in week 41+0 to ensure the ongoing pregnancy being uncomplicated), the trial was stopped early, using total mortality (not knowing if all cases of mortality is preventable) e.g, and not least this is of paramount importance to remember when forming the conclusion – it is not the case in this present paper neither in the conclusion.

Regarding the Apgar score, there a several studies showing that measures of Apgar 5 has association with long-term adverse outcomes. Further, when two different cut-points (close cut-points on rare outcomes) reach different results and may be that one measure to provide two different results of this analysis – one should be cautious as it reveals that the robustness of the calculated result is week. The discussion do not highlight this issue and the conclusion neither. Before we call Apgar 5 “softer” than Apgar 3 (as the authors do) we need to know which components of the scores the newborn were lacking (there is quite a difference depending on lacking tonus, respiration or heart activity) I suppose the authors do not have the exact information on the scores. I agree with the authors that Apgar score is not a good indicator of asphyxia making it even more hard to say what Apgar score contribute with in the overall composite score – is it just measuring the same as other included measures in the composite score?

When doing a meta-analysis one need to make sure the participants fulfill the inclusion criteria – this a strict rule applied to all meta-analysis. When discussing inclusion regarding gestational age (see also other reviewer suggesting to include more studies having less strict inclusion criteria) – the authors push back the argument as they want a strict focus on RCT looking at 41 vs 42. When it comes to study population the authors seem to care less about being strict in inclusion criteria. If we study low risk pregnant women we need to make sure that we do not include participant with a clearly complicated pregnancy at the time of inclusion. As no systematic evaluation with ultrasound was make before inclusion in the study we will experience a population with both complicated and uncomplicated pregnancies thus not all pregnancies included are uncomplicated pregnancies (this would need to be highlighted in the discussion and taken into account in the conclusion). However, those included in the Stockholm center had an ultrasound scan in week 41+0 and fulfilled the inclusion criteria. This is a very important information as no mortality was found among those included in the Stockholm center perhaps due to only including uncomplicated pregnancies. This means that your results may be due to studying a mixed population of low-risk and high-risk women and not due to the intervention studied. And I wonder why you do not have the same strict inclusion criteria as when you include your studies in the metaanalysis, why?

As suggested earlier, you could either make a major revision and provide a better transparency in the results, more coherent in your methodology, discussion, and conclusion of the study or the more correct methodological choice include only the Stockholm center from the Swepis (as you then do not violate the inclusion criteria of the meta-analysis studying only uncomplicated pregnancies).

Regarding the exposure, I do know it may be hard to be more specific – but the statement is very generalized – would you accept this in a trial with medication (a regime compared with another regime not very well described or two different operation procedures – one being generalized to a many different procedures with no further indication of the most used method). As we do not have more specific information on the regime of monitoring one should again discuss this more properly and be very cautious when forming the conclusion. If you include only the Stockholm center – you will be able to state the exposure more clearly

How do the author find it acceptable to compare with the arrive trial – a trial in which induction was made in week 39 (you at the same time argue that you have very strict criteria on which studies you are able to include as therefor studies with only few days differences are left out) But you find it okay to compare with Arrive trial where induction is in week 39 instead of week 41 as your study. Further, it is well-known in the clinical field (unfortunately not stated in the paper) that in the Arrive trial – those enrolled in the intervention group (induction in week 39) were many places cared for by midwives until birth while those enrolled in the reference group were care for by a mix of physicians and midwives which is normal practice in USA. We do know that there is an association between provider and cesarean section – that why it is of paramount importance to state a transparent description of the regime the participant receive (same as with your monitoring regime – ultrasound or not ultrasound)

It is sad to discover that the most common side effect of the intervention is not studied due to lack of coding – one could collected the data besides the official coding – would we consider this to be ethical correct if it was an operation we were studying? I do know you can´t change that now – but at least being more transparent about it would be admirable. Of course cesarean section and asphyxia may be related with hyperstimulation as you state. But hyperstimulation is a very severe and uncomfortable situation for the birthing women – very painful and there are also high risk scenarios related to the situation such as uterus rupture, bleeding, and placenta abruption (not mentioned in the paper). All situations where the women suffer a great deal and it may even affect her reproductive health in the future. In your conclusion you advocate for women to choose an induction based on the very uncertain estimate on the rare outcome mortality in this study (with several methodological limitation not made transparent in the paper)– with no mention of some of the other side-effects of the treatment. Further, decision-making is not always easy especially not in the end of a pregnancy and you provide no literature on this complex situation in you introduction or discussion but feel free to recommend women being offered induction in 41 without having own or other data to support this. Actually, paper published about decision-making on induction of labour in late pregnancy shows an overwhelming lack of transparency of information, involving in decision-making of the women – so how can you conclude the statement on women being offered induction in 41?

In general, the present meta-analysis seem to:

- Report a selected perspective on state of the art literature in the field (not very balanced)
- Not being transparent in methodological choices and how these affect the results
- Always make strict choices to follow a path towards a results favoring induction instead of being coherent with reflection regarding solid methodological and clinical choices
- Not being reasonable in making a sound conclusion (forget limitations on how data were handled and calculated and draw conclusions without data to back-up the conclusion)

There are already enough papers being more position-based than evidence-based within induction of labour research – we don’t need more of them. We need sound and relevant papers as it is an area that have already being handled not very wisely and the pregnant women are those paying the price. But also the women may end up losing confidence in health care professionals especially those bringing forward positions (on either side) rather than science (and denied an induction when advised and medical indicate).

In the obstetric field we have too many of these examples (confirm our positions rather than making solid science) and we do not need to continued down this path – we (the obstetric field) were awarded the “wooden spoon” by Archie Cochrane long time ago as we made to much treatment with no or minor effect and did not implement treatment with effect – we still need to make an effort to be more scientific.
