# Supplementary material for: Induction of labour at 41 weeks or expectant management until 42 weeks: A systematic review and an individual participant data meta-analysis of randomised trials
Source: PLoS Med. 2020 Dec 8;17(12):e1003436. doi: 10.1371/journal.pmed.1003436 (PMC7723286; doi:10.1371/journal.pmed.1003436)
Supplement: Attachment — Submitted filename: Reply to reviewer #2 final.docx [file pmed.1003436.s015.docx]

Review of the Manuscript D-20-03510 for Plos Medicine

Thank you for the opportunity to review the revised revision of Manuscript D-20-03510

Overall, the authors have not addressed the comments and suggestions or provided a relevant argumentation for not addressing the limitations of the paper. In the present form the paper is more a position paper than a scientific paper. Please find my elaboration below

We respect your opinion but disagree. We have, as stated in our previous reply, executed this research in line with existing guidelines and research custom. We have addressed in our introduction and discussion preexisting research, both of conflicting results and of evidence in line with our findings.

I still do not understand why the authors do not include all relevant body of evidence in their introduction. The authors will not include the meta-analysis published by Rydahl et al. because they also have measures on pregnancies extending 42+0. But the authors includes the Cochrane review with several studies including Hannahs trial (which is making a huge contribution to the final result of the meta-analysis) but quite a few of the studies in the Cochrane review examine pregnancies extending beyong 42+0.

We have previously explained that we focused on RCT’s and not on other designs because: 1) We aimed to compare two well defined **management strategies**, it is not a comparison of two ways of actual start of labor as in cohort studies. To explore what will happen after **intended** elective induction and after **intended** expectant management, inclusion of RCT studies is the only option. 2) Furthermore, cohort studies are prone for selection bias, in a RCT the management strategies are randomly assigned to participants.

We have added a sentence in the manuscript explaining why the overall outcome of the Cochrane review is probably not applicable to the 41-42 weeks’ timeframe: “The conclusion is based on all included studies of the systematic review but a majority of the included trials had expectant management groups that allows expectant management until far beyond 42 weeks, which could have influenced the overall outcomes.”

We also added a sentence regarding not including other studies than RCT’s: “Furthermore, non-experimental studies without clear randomisation procedure are prone for selection bias. Also, we aimed to evaluate outcomes of intended management strategies, not actual start of labour, because women have to decide on a management strategy before they know if and when they will go into spontaneous onset of labour. For this reasons we only included RCT’s in our IPD-MA.”

Further, another recent national Danish study by Rydahl (Denmark have a comparable setting to Sweden) examined a new national regime of earlier induction of labour in week 41 found no benefit in mortality using a robust method (interrupted time-series and Poisson regression) when studying rare outcomes (which is quite challenging to study in RCT). You have also experienced this core issue in the SWEPIS trial as you had to stop the trial earlier and ending up with overestimation of mortality in your study (your national Swedish numbers of mortality are lower compared to what you found in your trial).

We have read with interest the Rydahl et al., 2019 paper you are referring to and we also referred to it in our discussion section of our paper. This registry study shows interesting results emphasizing the need for RCT’s with clear comparison of management strategies in order to study causality because if induction will do more harm than good or has indeed too little advantage with major unwanted side effects obstetric policy should be reconsidered. We also referred to a study of Zizzo et al., 2017 that analysed almost the same population with an opposite conclusion than Rydahl. Furthermore, we also referred to Pyykonen et al., 2018, Grunewald et al., 2011 and Lindegren et al., 2017, all cohort studies reporting on the effect of policy change regarding induction of labor in low risk women with overdue pregnancies.

Regarding the early stopping of SWEPIS please see below.

Even though your are aware of this overestimation which is quite logic when you stop trials early on rare outcomes (due to fluctuate pattern) and you have also provided a sentence about it in your manuscript (elaboration of the fact that your Swedish national number are lower would have be admirable to include in the paper). Further, you forget this fact when making the study conclusion. You do not have valid data to make the conclusion stated in the manuscript – you would need to make a less conclusive conclusion.

One can start a discussion on which studies stopped early that may be affected by overestimation but in this case we have numbers showing that the Swepis trial have overestimated the mortality as we can compare with the national data. The authors need to move forward and not only discuss it but also relate to the actual numbers – this is not done in the study and the authors hold back important information to the readers.

We do agree that the perinatal mortality rate in the expectant management group (0.4%) was higher than expected. However, in a report from the Swedish National Board of Health and Welfare unexplained differences were found in stillbirth rates in different regions in Sweden. Sweden has a rather high stillbirth rate (3.7/1000) compared to other Nordic and many European countries and there has been no decline in stillbirth rates during the latest years in Sweden. Further, in another report from the Swedish National Board of Health and Welfare in 2018 the stillbirth rate was 0.2% at 41 gestational weeks in Sweden. However, to our knowledge, the perinatal mortality rate for the same timeframe as in SWEPIS has not been published. In a trial, such as SWEPIS, although large, but with few events concerning perinatal death, a few more deaths may well result in these figures. In addition, the way data on stillbirths and neonatal mortality are obtained in cohort studies and reports may not be quite comparable with how it is obtained in RCTs. The data on stillbirths in a retrospective cohort study represent register data and in a RCT the data including circumstances is validated in medical records.

We do not consider differences in outcome measures in our study to be caused by a flawed study design. As regards the remark on the early stopping of the SWEPIS trial, we want to state that though the magnitude of the risk of perinatal mortality is still not clear, it is clear from many studies comparing time frames that the trend of the risk of perinatal mortality and morbidity will not go in the opposite direction.

The author reply in their review comments “, in the Results section we already stated that: “It had some problems regarding study limitations for the outcome perinatal mortality.” it is good that the authors stated this in the results section. However, in a scientific paper one also need to discuss the limitation with transparency and clear out how these limitations influence the results. In this case, the limitations (including participants in the study without making sure they are low-risk (to be fair those in the Stockholm center – a part of the trial had a scan in week 41+0 to ensure the ongoing pregnancy being uncomplicated), the trial was stopped early, using total mortality (not knowing if all cases of mortality is preventable) e.g, and not least this is of paramount importance to remember when forming the conclusion – it is not the case in this present paper neither in the conclusion.

We do feel that we have discussed the shortcomings of our IPD-MA. We did address the different strategies regarding sonographic assessment and we perceive that we are more cautious than the reviewer, when interpreting the relevance of such assessment. As we wrote in our paper, there is insufficient evidence for an ultrasonographic assessment affecting the perinatal outcome in this population.

Our conclusion is a call to obstetrical caregivers to inform women on the uncertainty of the magnitude of perinatal mortality and to inform women with absolute risks (risk differences) instead of relative risks and to take parity into account in the counseling because we found no increased risk for multiparous women and a small increased risk of adverse perinatal outcome for nulliparous women. We do not state that women should be induced as the reviewer is clearly thinking. On the contrary, we provide the input for a nuanced picture with room to support women when they want to await spontaneous onset of labor until 42 weeks. In our view it is the woman that decides what management strategy suits her best, not the caregiver. This because, as the reviewer will recognize, there are more determinants that influences women when deciding on the strategy in late-term pregnancy, like the presence or absence of feelings of anxiety, physical condition, women’s own risk perception and way of life, less or more trust in natures course, preference for natural birth or hospital birth, previous birth experience etcetera.

Regarding the Apgar score, there a several studies showing that measures of Apgar 5 has association with long-term adverse outcomes. Further, when two different cut-points (close cut-points on rare outcomes) reach different results and may be that one measure to provide two different results of this analysis – one should be cautious as it reveals that the robustness of the calculated result is week. The discussion do not highlight this issue and the conclusion neither. Before we call Apgar 5 “softer” than Apgar 3 (as the authors do) we need to know which components of the scores the newborn were lacking (there is quite a difference depending on lacking tonus, respiration or heart activity) I suppose the authors do not have the exact information on the scores. I agree with the authors that Apgar score is not a good indicator of asphyxia making it even more hard to say what Apgar score contribute with in the overall composite score – is it just measuring the same as other included measures in the composite score?

We already have addressed this question in our last reply. We included only the most relevant severe adverse perinatal outcomes in the primary outcome and a 5-minute Apgar score of 0–3 correlates with neonatal mortality in large populations according the ACOG Committee Opinion no. 664 on Apgar score, therefore we considered this more robust than the 5-minute Apgar score <7. In Table 4 with perinatal outcomes we also show the primary outcome including 5-minute Apgar score <7 instead of <4. We don’t see how a primary outcome including 5-minute Apgar score <4 with a RR of 0.43 (0.21 to 0.91) can be seen as a sign of weak robustness in comparison with a primary outcome including 5-minute Apgar score <7 which has a RR of 0.65 (0.43.to 1.00).

We replaced “can be considered as a non specific sign of illness, which may be one of the first indications of encephalopathy” with “is associated with an increased risk on long term adverse neonatal outcome” in the method section. We also added a sentence int he discussion to clarify: ” because it is associated with an increased risk of long term adverse neonatal outcome”.

When doing a meta-analysis one need to make sure the participants fulfill the inclusion criteria – this a strict rule applied to all meta-analysis. When discussing inclusion regarding gestational age (see also other reviewer suggesting to include more studies having less strict inclusion criteria) – the authors push back the argument as they want a strict focus on RCT looking at 41 vs 42. When it comes to study population the authors seem to care less about being strict in inclusion criteria. If we study low risk pregnant women we need to make sure that we do not include participant with a clearly complicated pregnancy at the time of inclusion. As no systematic evaluation with ultrasound was make before inclusion in the study we will experience a population with both complicated and uncomplicated pregnancies thus not all pregnancies included are uncomplicated pregnancies (this would need to be highlighted in the discussion and taken into account in the conclusion). However, those included in the Stockholm center had an ultrasound scan in week 41+0 and fulfilled the inclusion criteria. This is a very important information as no mortality was found among those included in the Stockholm center perhaps due to only including uncomplicated pregnancies. This means that your results may be due to studying a mixed population of low-risk and high-risk women and not due to the intervention studied. And I wonder why you do not have the same strict inclusion criteria as when you include your studies in the metaanalysis, why?

The reviewer stated that the SWEPIS trial included low- and high risk women since ultrasound was not performed routinely in all participants of the trial and thus women with increased risk of adverse outcomes were included. However, inclusion in both trials was restricted to low risk women, which can be concluded from the in- and exclusion criteria. Current guidelines in Sweden do not include a routine ultrasonographic assessment at 41 weeks of gestation in order to define a low risk pregnancy. In The Netherlands routine ultrasonographic assessment is not yet included in the national guideline, but is in implemented in most local protocol agreements of local gynaecologists and midwifery practices. We are, however, aware that growth restricted fetuses are at increased risk of perinatal death but it is also clear from Divon et al., 1998 that only a minority (about 20 %) of stillbirths at late term and post term pregnancies are SGA, (in SWEPIS only one of six cases of perinatal death was SGA). Therefore, it cannot be claimed that a 41 week ultrasonographic scan would prevent most of the perinatal deaths occurring after 41 weeks. As stated above, the evidence for an ultrasonographic assessment at 41 weeks would reduce perinatal mortality is insufficient. In Lindqvist et al., 2014, a retrospective cohort study (not randomised), the perinatal adverse outcome (including perinatal death) was not significantly lower in the group with routine scan at 41 weeks compared with indicated scans (routine in most Swedish centers). Unfortunately, we still lack a fetal monitoring technique that effectively predicts and prevents stillbirths in late term/post term pregnancies.

As suggested earlier, you could either make a major revision and provide a better transparency in the results, more coherent in your methodology, discussion, and conclusion of the study or the more correct methodological choice include only the Stockholm center from the Swepis (as you then do not violate the inclusion criteria of the meta-analysis studying only uncomplicated pregnancies).

Regarding the exposure, I do know it may be hard to be more specific – but the statement is very generalized – would you accept this in a trial with medication (a regime compared with another regime not very well described or two different operation procedures – one being generalized to a many different procedures with no further indication of the most used method). As we do not have more specific information on the regime of monitoring one should again discuss this more properly and be very cautious when forming the conclusion. If you include only the Stockholm center – you will be able to state the exposure more clearly

Both SWEPIS and the INDEX trial are pragmatic trials, i.e. both trials are customised to the clinical setting in the respective country. Hence, IOL and expectant management are performed according to local clinical protocols. Therefore, the trials probably reflect better how it would turn out in the clinical setting if a change in policy would be the result of the trials. Regarding the comparison with RCTs evaluating medications or surgical techniques, one common objection regarding these kind of RCTs is that they are so strictly in their in- and exclusion criteria that they do not reflect the real clinical setting. Hence, when implemented the outcome of interest does not reflect the trial results.

How do the author find it acceptable to compare with the arrive trial – a trial in which induction was made in week 39 (you at the same time argue that you have very strict criteria on which studies you are able to include as therefor studies with only few days differences are left out) But you find it okay to compare with Arrive trial where induction is in week 39 instead of week 41 as your study. Further, it is well-known in the clinical field (unfortunately not stated in the paper) that in the Arrive trial – those enrolled in the intervention group (induction in week 39) were many places cared for by midwives until birth while those enrolled in the reference group were care for by a mix of physicians and midwives which is normal practice in USA. We do know that there is an association between provider and cesarean section – that why it is of paramount importance to state a transparent description of the regime the participant receive (same as with your monitoring regime – ultrasound or not ultrasound)

We mentioned in our previous answer the ARRIVE trial just as an example of one recent large randomised controlled trial comparing mode of delivery in low risk nulliparous women. We wanted to refer to a trial that compared women who underwent labor induction with those who had the alternative of expectant management, not with those who had a spontaneous onset of labour. We agree that there are limitations in the ARRIVE trial, such as the inclusion of a population of women not being representative of the general US population since it comprised young women (mean age 24 vs 23 years) and the rate of women of white ethnicity was lower than in the general US population (43% vs 45%). However, we found it interesting to mention the rate of caesarean delivery in the ARRIVE study in comparison with the rate of cesarean delivery in nulliparous women in our study to point out that we think that our population consisted of low risk women with an uncomplicated pregnancy.

It is sad to discover that the most common side effect of the intervention is not studied due to lack of coding – one could collected the data besides the official coding – would we consider this to be ethical correct if it was an operation we were studying? I do know you can´t change that now – but at least being more transparent about it would be admirable. Of course cesarean section and asphyxia may be related with hyperstimulation as you state. But hyperstimulation is a very severe and uncomfortable situation for the birthing women – very painful and there are also high risk scenarios related to the situation such as uterus rupture, bleeding, and placenta abruption (not mentioned in the paper). All situations where the women suffer a great deal and it may even affect her reproductive health in the future. In your conclusion you advocate for women to choose an induction based on the very uncertain estimate on the rare outcome mortality in this study (with several methodological limitation not made transparent in the paper)– with no mention of some of the other side-effects of the treatment. Further, decision-making is not always easy especially not in the end of a pregnancy and you provide no literature on this complex situation in you introduction or discussion but feel free to recommend women being offered induction in 41 without having own or other data to support this. Actually, paper published about decision-making on induction of labour in late pregnancy shows an overwhelming lack of transparency of information, involving in decision-making of the women – so how can you conclude the statement on women being offered induction in 41?

Again, we do not state that women should be induced as the reviewer suggests, we state women should be informed on all aspects of both management strategies, taking into account the uncertainty on perinatal mortality and according to parity, that is something completely different. A woman should decide for herself what management strategy suits her best. From previous reply: “because, as the reviewer will recognize, there are more determinants that influences women when deciding for a strategy in late-term pregnancy, like the presence or absence of feelings of anxiety, physical condition, women’s own risk perception and way of life, less or more trust in natures course, preference for natural birth or hospital birth, previous birth experience etcetera.”

We expect to publish soon results of women’s experiences of both the INDEX and SWEPIS study.

In general, the present meta-analysis seem to:

- Report a selected perspective on state of the art literature in the field (not very balanced)
- Yes, selective in the way that we compared trials evaluating the management strategies elective induction at 41 and at 42 weeks, as this was the subject of the debate. The best way to evaluate the effect of a strategy is to include trials not cohort studies or quasi-experimental studies or other designs which are prone to introduce selection bias.
- Not being transparent in methodological choices and how these affect the results
- As stated above, we do not agree with this statement. We have been totally transparent in our choices in our view and we discussed the limitations of our study.
- Always make strict choices to follow a path towards a results favoring induction instead of being coherent with reflection regarding solid methodological and clinical choices –
- We disagree with this comment. We have done two pragmatic trials reflecting the clinical situation in Sweden and The Netherlands at the time.We wanted to compare two clinical routines, induction at 41 weeks compared with expectant management and induction at 42 weeks. The potential benefits of an ultrasound in prolonged pregnancies are not enough studied (there are no RCTs) and we still lack evidence for a fetal monitoring technique that is effective to predict and prevent stillbirth in prolonged pregnancies. We therefore accepted in our study protocols that there were different routines for surveillance of prolonged pregnancies, furthermore there were different protocols for induction at the participating centres, thus reflecting the “real world” situation.
- The individual trials were started because of the hope to solve the strategy problem in late term pregnancy. In the INDEX trial (being a non-inferiority trial) it was expected (and hoped for) to find no difference in adverse outcomes in these low risk populations. Also for this review we regret that the outcomes weren’t better. But as researchers we have to accept the results, also if they don’t meet expectations.
- Not being reasonable in making a sound conclusion (forget limitations on how data were handled and calculated and draw conclusions without data to back-up the conclusion) –
- Unfortunately we disagree once again. Please find our response in this document.

There are already enough papers being more position-based than evidence-based within induction of labour research – we don’t need more of them. We need sound and relevant papers as it is an area that have already being handled not very wisely and the pregnant women are those paying the price. But also the women may end up losing confidence in health care professionals especially those bringing forward positions (on either side) rather than science (and denied an induction when advised and medical indicate).

In the obstetric field we have too many of these examples (confirm our positions rather than making solid science) and we do not need to continued down this path – we (the obstetric field) were awarded the “wooden spoon” by Archie Cochrane long time ago as we made to much treatment with no or minor effect and did not implement treatment with effect – we still need to make an effort to be more scientific.

The researchers of our team have the starting point that trust in- and protecting of normal birth should be promoted, but we don’t want to close our eyes for unwelcome outcomes. Our research meets the standards of scientific integrity.
